# Sequence analysis of the hepatitis D virus across genotypes reveals highly conserved regions amidst evidence of recombination

**DOI:** 10.1093/ve/veaf012

**Published:** 2025-02-27

**Authors:** Shruti Chowdhury, Carina Jacobsen, Daniel P Depledge, Heiner Wedemeyer, Lisa Sandmann, Helenie Kefalakes

**Affiliations:** Department of Gastroenterology, Hepatology, Infectious Diseases and Endocrinology, Hannover Medical School, Carl-Neuberg-Str. 1, Hannover 30625, Germany; Cluster of Excellence RESIST, EXC-2155, Hannover Medical School, Carl-Neuberg-Str. 1, Hannover 30625, Germany; Department of Gastroenterology, Hepatology, Infectious Diseases and Endocrinology, Hannover Medical School, Carl-Neuberg-Str. 1, Hannover 30625, Germany; Cluster of Excellence RESIST, EXC-2155, Hannover Medical School, Carl-Neuberg-Str. 1, Hannover 30625, Germany; Cluster of Excellence RESIST, EXC-2155, Hannover Medical School, Carl-Neuberg-Str. 1, Hannover 30625, Germany; German Center for Infection Research (DZIF), Partner Site Hannover-Braunschweig, Hannover 30625, Germany; Institute of Virology, Hannover Medical School, Carl-Neuberg-Str. 1, Hannover 30625, Germany; Department of Gastroenterology, Hepatology, Infectious Diseases and Endocrinology, Hannover Medical School, Carl-Neuberg-Str. 1, Hannover 30625, Germany; Cluster of Excellence RESIST, EXC-2155, Hannover Medical School, Carl-Neuberg-Str. 1, Hannover 30625, Germany; German Center for Infection Research (DZIF), Partner Site Hannover-Braunschweig, Hannover 30625, Germany; D-SOLVE Consortium, an EU Horizon Europe funded project (No 101057917), Hannover 30625, Germany; Department of Gastroenterology, Hepatology, Infectious Diseases and Endocrinology, Hannover Medical School, Carl-Neuberg-Str. 1, Hannover 30625, Germany; Cluster of Excellence RESIST, EXC-2155, Hannover Medical School, Carl-Neuberg-Str. 1, Hannover 30625, Germany; German Center for Infection Research (DZIF), Partner Site Hannover-Braunschweig, Hannover 30625, Germany; D-SOLVE Consortium, an EU Horizon Europe funded project (No 101057917), Hannover 30625, Germany; Department of Gastroenterology, Hepatology, Infectious Diseases and Endocrinology, Hannover Medical School, Carl-Neuberg-Str. 1, Hannover 30625, Germany; Cluster of Excellence RESIST, EXC-2155, Hannover Medical School, Carl-Neuberg-Str. 1, Hannover 30625, Germany; German Center for Infection Research (DZIF), Partner Site Hannover-Braunschweig, Hannover 30625, Germany; D-SOLVE Consortium, an EU Horizon Europe funded project (No 101057917), Hannover 30625, Germany

**Keywords:** genotypes, reference sequences, hepatitis D virus, epitopes, recombination

## Abstract

Sequence diversity of the hepatitis D virus (HDV) may impact viral clearance, contributing to the development of chronic infection. T-Cell-induced selection pressure and viral recombination can induce diversity throughout the viral genome including coding and noncoding regions, with the former potentially impacting viral pathogenicity and the latter exerting regulatory functions. Here, we aim to assess sequence variations of the HDV genome within and across HDV genotypes. Sequences from 721 complete HDV genomes and 793 large hepatitis D antigen (L-HDAg) regions belonging to all eight genotypes and published through December 2023 were compiled. Most retrieved sequences belonged to Genotype 1, whereas for Genotype 8, the fewest sequences were available. Alignments were conducted using Clustal Omega and Multiple Alignment using Fast Fourier Transform. Phylogeny was analysed using SplitsTree4, and recombination sites were inspected using Recombination Detection Program 4. All reported sequences were aligned per genotype to retrieve consensus and reference sequences based on the highest similarity to consensus per genotype. L-HDAg alignments of the proposed reference sequences showed that not only conserved but also highly variable positions exist, which was also reflected in the epitope variability across HDV genotypes. Importantly, *in silico* binding prediction analysis showed that CD8^+^ T-cell epitopes mapped for Genotype 1 may not bind to major histocompatibility complex class I when examining their corresponding sequence in other genotypes. Phylogenetic analysis showed evidence of recombinant genomes within each individual genotype as well as between two different HDV genotypes, enabling the identification of common recombination sites. The identification of conserved regions within the L-HDAg allows their exploitation for genotype-independent diagnostic and therapeutic strategies, while the harmonized use of the proposed reference sequences may facilitate efforts to achieve HDV control.

## Introduction

The hepatitis D virus (HDV) is a small pathogenic enveloped virus and the causative agent of the most severe form of chronic viral hepatitis. Its genome is a circular negative-sense single-stranded RNA (ssRNA), with a length of ∼1700 nucleotides. As one of the smallest RNA genomes in all known animal viruses (Negro and Lok 2023), the HDV genome encodes a single protein, the hepatitis D antigen (HDAg). The HDAg exists in two isoforms: the small HDAg (S-HDAg; mostly 194-195 amino acids in length) and the large HDAg (L-HDAg; mostly 213-215 amino acids in length), with the L-HDAg resulting from enzymatic editing of the S-HDAg amber stop codon, leading to addition of 19 or 20 amino acids, which is dependent on the HDV genotype (Casey et al. 1992). While the S-HDAg is necessary for HDV’s replication, the L-HDAg suppresses replication and, through interaction with the hepatitis B surface antigen, is essential for its envelopment and spread (Lee et al. 1995).

There are currently eight HDV genotypes exhibiting distinct geographical distributions and having varying impacts on HDV infection course, clinical outcome, and treatment response (Chen et al. 2019). It has been demonstrated that Genotype 1 is present in most parts of the world, where it has been associated with the development of liver complications, such as liver cirrhosis and hepatocellular carcinoma (Wranke et al. 2018). Genotypes 2 and 4 mostly localize in East and South-East Asia and are associated with milder disease courses (Wranke et al. 2018), while Genotypes 5–8 have a West and Central African origin. Infection with Genotype 5 shows not only better treatment response to interferon (Spaan et al. 2020) but also a higher risk for the development of cirrhosis (Roulot et al. 2020). By contrast, Genotype 3, prevalent in South America, often leads to acute liver failure (Wranke et al. 2018). Currently, the published reference sequence for Genotype 1 is among the first HDV sequences that had been investigated (Makino et al. 1987, Saldanha et al. 1990). For the remaining genotypes, provisional reference sequences are available from the National Center for Biotechnology Information (NCBI) Viral RefSeq Project (ID PRJNA485481), and reference sequences had additionally been suggested by Miao *et al*. in 2018, by analysing a more limited dataset (Miao et al. 2018). However, provision of the most representative reference sequences for all genotypes, based on the current dataset, will allow to streamline the efforts to achieve HDV control.

Since HDV is a satellite virus of the hepatitis B virus (HBV), its tropism is confined to human hepatocytes, as the HDV nucleoprotein complex interacts with the HBV envelope. Recently though, more HDV-like agents were discovered in vertebrates and invertebrate species, harbouring replication in other organs than the liver (Gnouamozi et al. 2024) and suggesting that the L-HDAg may have evolved during adaptation of HDV to the human host. In RNA viruses, recombination is considered as one of the mechanisms used to expand the viral host range (Brown 1997, Gibbs and Weiller 1999). It is considered to occur more often during chronic viral infections, as a single host has increased chances of acquiring mixed infections (Simon-Loriere and Holmes 2011), and it may also contribute to evasion of host immunity (Malim and Emerman 2001). While it has been considered that recombination can purge deleterious mutations or create advantageous genotypes, in HDV infection, it has been suggested to be a by-product of its RNA structure and host polymerase-driven replication (Chao et al. 2017). A recent study found a considerable rate of recombination between full-length genomes of HDV from Central Asia (Bahoussi et al. 2022), while there is also evidence of recombination within and between some HDV genotypes (Wang and Chao 2005, Lin et al. 2017, Miao et al. 2018). The current analysis takes into account the highest number of published full-length genome sequences to get a broader overview on the presence of recombination within and across HDV genotypes.

HDAg-derived epitopes recognized by CD8^+^ T cells play a critical role in the immune response against HDV by facilitating recognition and elimination of infected cells (Kohsar et al. 2021). Sequence diversity can affect epitope presentation and recognition, leading to reduced viral clearance and development of chronic infection. Recent studies have focused on Genotype 1–based epitopes, showing that T-cell selection pressure promotes viral escape (Karimzadeh et al. 2018, 2019, Kefalakes et al. 2019). However, whether the corresponding sequences of these epitopes are also presented by major histocompatibility complex class I (MHC-I) in the other HDV genotypes and whether epitope diversity may be facilitated by viral recombination have not been investigated. Since recombinant strains may lead to epitope variability and heightened virulence, affecting their spread within populations and subsequent response to treatment, understanding different aspects of epitope diversity will facilitate the development of targeted treatment options against HDV.

## Materials and methods

### Nucleotide and protein datasets

A total of 721 genomic sequences (1600–1700 nucleotides), covering the complete HDV genome, and 793 protein sequences (~214 amino acids), covering the L-HDAg were compiled from the HDV Database (HDVdb) (Usman et al. 2020) and from NCBI (Supplementary Table S1). HDVdb includes all HDV sequences published until April 2020 (Usman et al. 2020), and the majority of sequences were directly retrieved from here. Sequences that were published between April 2020 and December 2023 were retrieved from NCBI using the keywords ‘Hepatitis Delta Virus complete genome’ (filtering for sequence lengths of 1600–1800 nucleotides) and ‘Large Hepatitis Delta Antigen’ (filtering for sequence lengths of 209–219 amino acids). Extracted sequences included all eight genotypes with information on their countries of origin.

Most complete genome and L-HDAg sequences, included in this analysis, were obtained by either Sanger or short-read sequencing. However, 30 genomic consensus sequences were obtained by nanopore sequencing (Charre et al. 2023) and were deposited as raw sequence reads in the Sequence Read Archive (SRA) with BioProjectID PRJNA759204. Out of these, the L-HDAg was only retrieved from 20 genomic sequences after translation using Expasy Translate (v3.0) (Duvaud et al. 2021).

Of the 53 full-length nucleotide sequences belonging to Genotype 3, 41 sequences from Brazil were published in the coding sequence form, otherwise known as the antigenome (Miao et al. 2018). These sequences were converted to their corresponding genomic forms using Reverse Complement (v1, Sequence Manipulation Suite) (Stothard 2000). While most published genomic sequences have a standard reading site from 1 to 1682, some genomic sequences were found to have different starting and ending points. This was observed for 100 out of 480 Genotype 1 sequences from Turkey and Cameroon, 6 out of 19 sequences of Genotype 6, and 30 out of 51 sequences of Genotype 7, both from Cameroon. Before aligning these sequences, they were manually standardized to obtain starting and ending points from 1 to 1682 (Supplementary Table S2).

Genotype identification for published sequences with unreported genotypes was conducted based on their nucleotide sequences using HDVdb (Usman et al. 2020).

### Multiple sequence alignments

Consensus sequences for each genotype were obtained by performing multiple sequence alignments of the HDV genome sequences (Multiple Alignment using Fast Fourier Transform, MAFFT; v7.471) (Katoh and Standley 2013) through SnapGene (v6.2.2) (Weber et al. 2020). The threshold for collecting the consensus sequences was set to >50%. Consensus sequences were aligned with all sequences of their respective genotype to generate percentage identity matrices via MAFFT (v7.520) on the European Molecular Biology Laboratory-European Bioinformatics Institute web server (Katoh and Standley 2013, Madeira et al. 2022). Recombination analyses were performed on aligned sequences from MAFFT (v7.520) for each genotype separately and in comparative analyses (Katoh and Standley 2013, Kuraku et al. 2013).

Consensus sequences of the L-HDAg protein for each genotype were identified by SnapGene (v6.2.2) using Clustal Omega (v1.2.4) (Sievers et al. 2011, 2020, Sievers and Higgins 2018, Weber et al. 2020). L-HDAg sequences of the newly suggested nucleotide references were collected from NCBI. When functional L-HDAg protein sequences were absent, they were retrieved by manual translation, using Expasy Translate (v3.0) (Duvaud et al. 2021). Amino acid conservation across genotypes was evaluated by aligning the eight L-HDAg protein sequences and the consensus sequences, respectively, to the NCBI HDV reference protein sequence NP_597693.2. Multiple sequence alignments were conducted using Clustal Omega (v1.2.4) and visualized using SnapGene (v7.2) (Sievers et al. 2011, 2020, Sievers and Higgins 2018, Weber et al. 2020).

### CD8^+^ T-cell epitope variation within and across genotypes

Ten CD8^+^ T-cell epitopes, previously confirmed *in vitro*, were selected for their sequence comparison within and across genotypes (Supplementary Table S3) (Karimzadeh et al. 2018, 2019, Kefalakes et al. 2019). The published epitope sequences, L-HDAg reference sequence NP_597693.2, and the eight newly suggested reference sequences were analysed manually to identify the variations of amino acids across genotypes. Biochemical properties and variations within genotypes were visualized by frequency plots using Weblogo (v2.8.2) (Schneider and Stephens 1990, Crooks et al. 2004). CD8^+^ T-cell epitope-MHC-I binding affinities of epitope variants were determined *in silico* through the Immune Epitope Database and analysis resource (v2.28) (Vita et al. 2019). MHC-I binding predictions were assessed using the NetMHCpan BA algorithm (v4.1) (Reynisson et al. 2020). Percentile ranks and binding affinities of the epitopes were predicted based on their previously described corresponding Human Leukocyte Antigen (HLA) class I alleles (Karimzadeh et al. 2018, 2019, Kefalakes et al. 2019).

### Phylogenetic trees and recombination analyses

All 721 genomic sequences were used to create a circular phylogenetic tree showing segregation into the eight genotypes. The sequences were aligned in MAFFT (v7.520), and the aligned Newick file was used to visualize and annotate the tree on the Interactive Tree Of Life (iTOL v6.9.1) (Letunic and Bork 2007, Katoh and Standley 2013). Bootstrapping analysis was performed to confirm the branching of the tree nodes.

Evolutionary relationship between the eight suggested HDV reference sequences was evaluated by forming phylograms using MAFFT (v7.520) and visualized using Phylo.io (v1.0.0) (Katoh and Standley 2013, Kuraku et al. 2013, Robinson et al. 2016).

Neighbour-Net equal angle network trees were built with the aligned files of all sequences of the HDV genome to check for phylogenetic relationships and viral recombination within and between genotypes using SplitsTree4 (v4.19.1) (Huson and Bryant 2006). Tree reliability was confirmed after running bootstrapping analysis with 1000 replications.

Recombination Detection Program (RDP) 4 (v4.101) was used to identify breakpoints and sequences taking part in recombination using the aligned files for each HDV genotype (Martin et al. 2015). Statistical Tests for Detecting Gene Conversion, maximum chi-square, RDP, Bootscan, Chimaera, and Sister Scanning were the overall recombination-detecting algorithms used to record the events of recombination (Smith 1992, Padidam et al. 1999, Gibbs et al. 2000, Martin and Rybicki 2000).

## Results

### Collection of complete genome and L-HDAg sequences shows geographic distribution of HDV across the world

**Figure 1.**
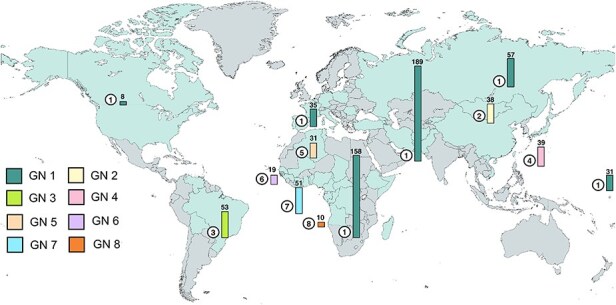
**Geographic distribution of HDV genotypes across the world**. Compilation of all countries where complete HDV genome and L-HDAg sequences were collected from until December 2023 (turquoise). The length of the bar graphs represents the amount of HDV genome sequences collected for each HDV genotype. Numbers in circles show the genotype. Two Genotype 1 genomic sequences have unknown origin and are thus not accounted for in the map.

A total of 721 complete HDV genome sequences and 793 L-HDAg protein sequences were retrieved from HDVdb (Usman et al. 2020), NCBI, and SRA (Supplementary Table S1). The sequences covered the eight known HDV genotypes, with 480 complete genome sequences and 596 L-HDAg sequences, available for Genotype 1, whereas less sequences were available for the other genotypes (Table 1). While Genotype 1 is spread ubiquitously around the world, the available sequences were originating mostly from Africa, as well as West and Central Asia (Fig. 1). A total of 158 of these sequences were compiled from various countries in Africa, especially Cameroon, whereas 189 sequences were attributed to countries primarily in Western Asia, such as Kyrgyzstan, Pakistan, and Iran (Fig. 1). Genotypes 2 and 4 are found primarily in East and Central Asia, Genotype 3 in South America, and Genotypes 5–8 across several countries of Africa. Genotype 8 found in West Africa had the least number of sequences recorded so far.

**Table 1.** Geographic regions and countries of origin of the compiled HDV sequences published until December 2023.

Genotype	Complete HDV genome sequences	L-HDAg protein sequences	Continents	Countries
1	480	596	Asia, Africa, Europe, North America, Oceania	Cameroon, Pakistan, Turkey, Russia, Taiwan, Mongolia, Kyrgyzstan, Vietnam, Kiribati, Iran, Israel, China, Tunisia, Angola, Democratic Republic of the Congo, Algeria, Madagascar, Cote d’Ivoire, Central African Republic, Ethiopia, Republic of the Congo, Guinea-Bissau, Togo, Chad, Nigeria, Somalia, Benin, Romania, Mayotte, Spain, France, Italy, Poland, Portugal, Germany, Armenia, Georgia, Lebanon, Afghanistan, Japan, Nauru, USA, Canada
2	38	23	Central, East and South-East Asia	Taiwan, Vietnam, Kyrgyzstan, Russia, Japan, China
3	53	44	South America	Brazil, Bolivia, Venezuela
4	39	39	East Asia	Taiwan, Japan
5	31	22	Africa	Guinea, Cote d’Ivoire, Mali, Togo, Guinea-Bissau, Senegal, Nigeria, Ghana, Portugal
6	19	17	Africa	Cameroon, Mali, Nigeria, Central African Republic, Cote d’Ivoire, Angola, Russia
7	51	47	Africa	Cameroon, Guinea, Spain
8	10	5	Africa	Republic of the Congo, Mali, Democratic Republic of the Congo, Cote d’Ivoire, Angola, Senegal, Gabon

Total frequencies of complete HDV genome sequences and L-HDAg protein sequences belonging to each genotype.

### Phylogenetic analysis of HDV sequences allows identification of most representative sequences per genotype

**Figure 2.**
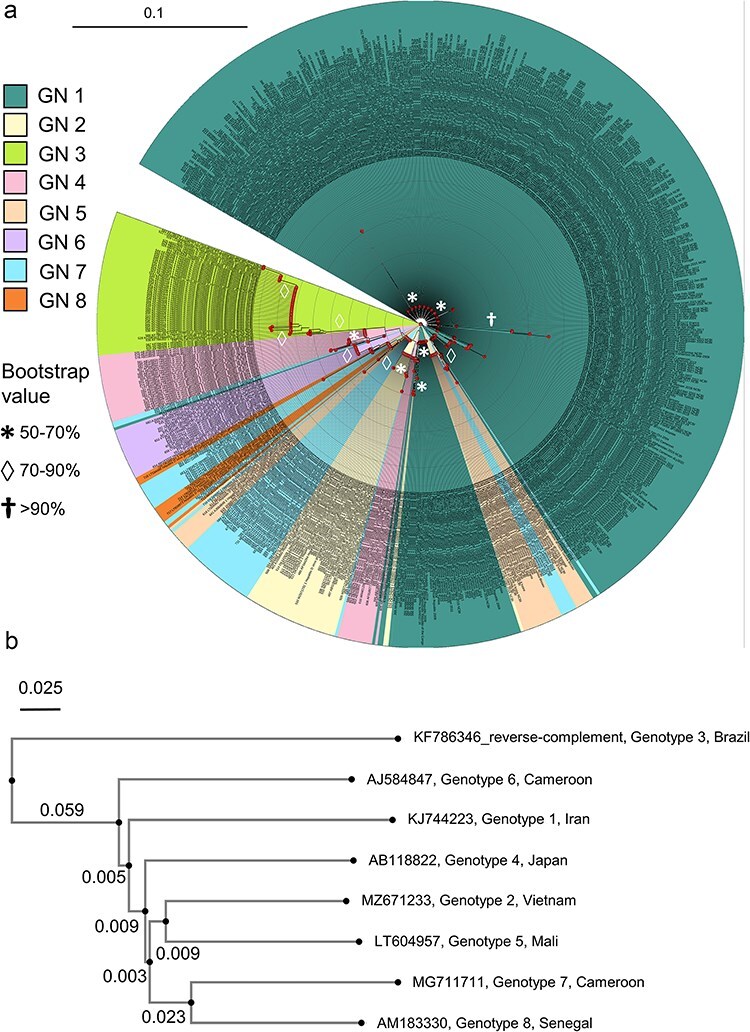
**Phylogenetic segregation of full-length HDV genome sequences and their evolutionary descent.** (a) Circular phylogenetic tree of 721 full-length HDV genome sequences. The sequences are colour-coded based on the genotypes. Circular dots indicate nodes of the phylogenetic tree. Bootstrapping values are indicated by symbols adjacent to the tree nodes—moderately supported: 50%–70%, well supported 70%–90%, very well supported: >90%. (b) Phylogenetic tree displaying the most representative reference sequences of the HDV genome. The sequence for Genotype 3 is reverse complementary to the published antigenomic sequence KF786346. The numbers over branches indicate the branch lengths with the tree scales indicating the number of nucleotide substitutions per site.

Phylogenetic analysis of the 721 full-length HDV genome sequences showed the frequency and the degrees of relatedness among the eight genotypes (Fig. 2a). Most HDV sequences belonged to Genotype 1 which originates from several nodes near the root of the tree. Sequences of certain genotypes such as 2 and 4 were also found to map separately in different sections of the tree depending on their subgenotype-level division. As visualized by the branching pattern and the relative depth of the nodes, one branch from the root diverged further into Genotype 2 and some sequences of Genotype 4, followed by further branching into Genotypes 7, 8 and 6. As Genotypes 2 and 4–8 were all connected by nodes closer to Genotype 1, this indicates that they are more closely related. By contrast, Genotype 3 clustered farthest away from the other genotypes with the most distant nodes from the root, and thus, it is more distantly related to the rest (Fig. 2a).

To provide reference sequences based on the analysis of all available HDV sequences reported until December 2023 (Table 1), the sequences of each genotype were grouped and aligned. MAFFT alignment for each genotype generated nucleotide consensus sequences, representative for each genotype (Supplementary Table S4). Percentage identity matrices constructed for each genotype helped to evaluate the complete HDV genome sequence closest to each consensus. The sequences that had the maximum number of amino acids identical to each consensus, thus having the highest percentage identity, were proposed as the most representative or reference sequences for each genotype (Supplementary Tables S5 and S6). Here, all newly proposed reference sequences had a percentage identity of >92%. While the originally proposed reference sequence for Genotype 1 (NC_001653.2 from USA) had a percentage identity of 89.49%, the percentage identity of our newly proposed Genotype 1 reference sequence (KJ744223 from Iran) was 92.2% and thus higher than the original reference sequence. All new reference sequences also had higher percentage identities to the consensus when compared to the provisional sequences from the NCBI Viral RefSeq Project. For Genotype 8, the Provisional RefSeq AM183330 showed the highest percentage identity with the Genotype 8 consensus and therefore matched our proposed reference sequence (Supplementary Table S5). The proposed reference sequence of Genotype 2 arises from Vietnam, that of genotype 4 from Japan, while the reference sequence for Genotype 5 is derived from Mali; the reference sequences for both Genotypes 6 and 7 originate from Cameroon, and that of Genotype 8 from Senegal. Of note, the suggested genomic reference for Genotype 3 is the reverse complement of the HDV sequence KF786346 from Brazil, originally published in its antigenomic form.

The evolutionary relationships between the eight newly suggested HDV genome reference sequences were evaluated by a phylogram (Fig. 2b). The reference sequences for Genotypes 7 and 8 found in West and Central Africa mapped close to each other, whereas the sequences for Genotypes 2 and 5 derived from South East Asia and Africa clustered together. The Genotype 4 reference sequence originating from East Asia shared a most recent common ancestor with all the above, followed by Genotype 1. In addition, the reference sequence for Genotype 6 from Africa mapped farther away, followed by Genotype 3 from South America. The latter was found to be most dissimilar when compared to all other genotypes (Fig. 2a). Overall, systematic analysis of published HDV sequences allows us to identify the most representative sequences for each genotype.

### Sequence alignment of L-HDAg sequences shows conserved regions across genotypes

**Figure 3.**
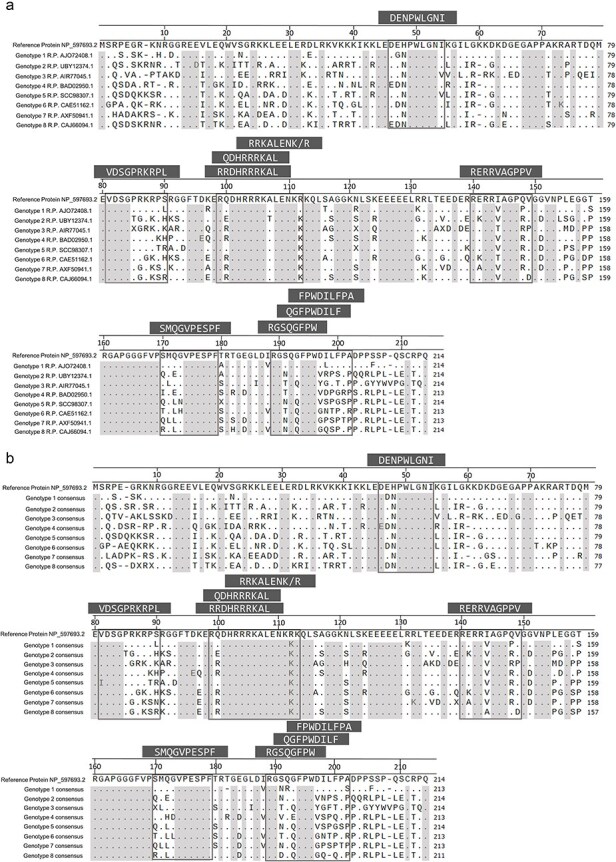
**Sequence alignment of the L-HDAg protein reference and consensus sequences showing amino acid conservation.** Regions highlighted in grey have a conservation of 90% or higher. Experimentally confirmed CD8^+^ T-cell epitopes are shown in dark grey boxes on top. (a) Amino acid conservation across the L-HDAg protein of the eight proposed genomic reference sequences with the original NCBI reference protein NP_597693.2 for Genotype 1. (b) Regions of similarity are shown across the L-HDAg consensus sequences obtained for each HDV genotype. The original NCBI reference protein NP_597693.2 for Genotype 1 has been used for comparison. Abbreviation: R.P., reference protein.

For protein sequence analysis, the eight L-HDAg sequences of the proposed nucleotide references were aligned with the NCBI HDV reference protein NP_597693.2. Here, several sections of the protein had identical amino acids that were >90% conserved across genotypes (Fig. 3a). Regions that were almost 100% conserved in the L-HDAg protein of the reference sequences included amino acid positions 49–53, 79–84, 101–106, 125–130, 160–169, and 174–179. The analysis also showed that ∼50% of the length of the L-HDAg sequences was completely conserved across genotypes. Additionally, L-HDAg consensus sequences were generated for each genotype by aligning all published L-HDAg sequences (Supplementary Table S7), obtaining similar results (Fig. 3b).

To further examine the viral epitopes present in the protein sequences, 10 previously confirmed CD8^+^ T-cell epitopes that had been mapped for Genotype 1 were selected for L-HDAg sequence comparison within and across genotypes (Karimzadeh et al. 2018, 2019, Kefalakes et al. 2019) (Fig. 3, Supplementary Fig. 1 and Supplementary Table S3). The published epitope sequences were compared to the original L-HDAg reference sequence NP_597693.2 and the eight newly proposed reference sequences. Epitopes present towards the amino terminus of the sequence were found to be more conserved across all genotypes with only one or two amino acid positions altering (Supplementary Table S8). By contrast, epitopes located towards the carboxy terminus of the L-HDAg sequence were found to be highly variable across genotypes (Supplementary Table S9).

To understand the epitope variations occurring within each genotype, frequency plots were generated on WebLogo. Conserved epitopes had more identical amino acids within the same genotype (Supplementary Fig. S2). By contrast, the nonconserved epitopes had a lot more variation in amino acid composition within an individual genotype (Supplementary Fig. S3). Furthermore, noticeable differences were observed in the biochemical composition of the amino acids. While conserved epitopes consisted of mostly basic amino acids, the majority of the epitopes in the hypervariable region were composed of hydrophobic and polar amino acids (Supplementary Figs S2 and S3).

To check binding between the epitope variants present in the different genotypes with the published MHC-I alleles, *in silico* binding prediction analysis was performed (Supplementary Tables S8 and S9). Some epitopes such as L-HDAg_104–112_ and L-HDAg_170–179_ had uniformly good binding scores across all HDV genotypes. Consistent binding to published MHC-I alleles was absent for specific epitopes mapped in Genotype 1, such as L-HDAg_99–108_ and L-HDAg_194–202_, when looking at the other genotypes. Specifically, epitope L-HDAg_99–108_ of Genotype 2 did not bind to the HLA-B*27:05 allele, while epitope L-HDAg_194–202_ of Genotype 4 had no binding to HLA-B*07:02. Moreover, only the Genotype 1 variable epitope L-HDAg_192–200_ binds to the proposed MHC-I allele. Of note, L-HDAg_81–90_ shows no *in silico* binding to the published MHC class I allele in any of the genotypes, confirming that *in silico* binding needs *in vitro* verification and that epitopes mapped for Genotype 1 may have abrogated binding in other HDV genotypes due to sequence variability.

### Viral recombination is observed within and across all HDV genotypes

**Figure 4.**
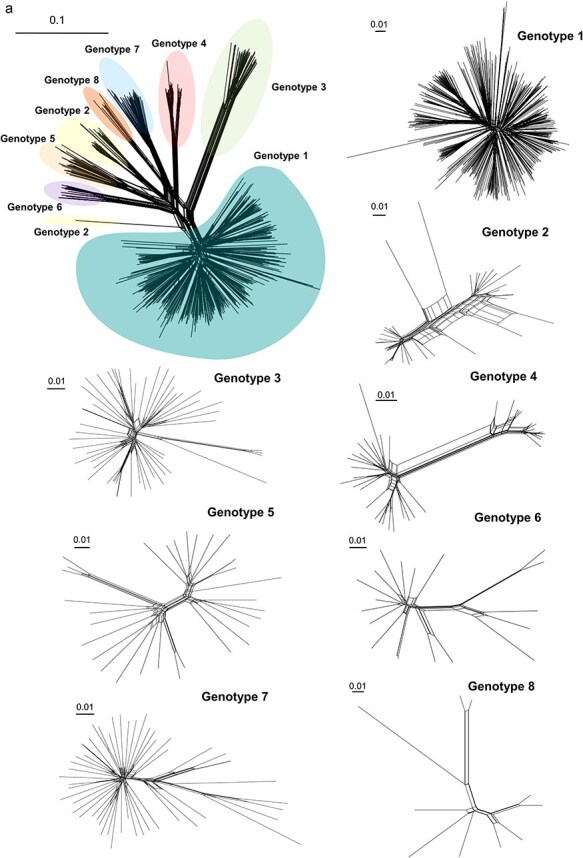

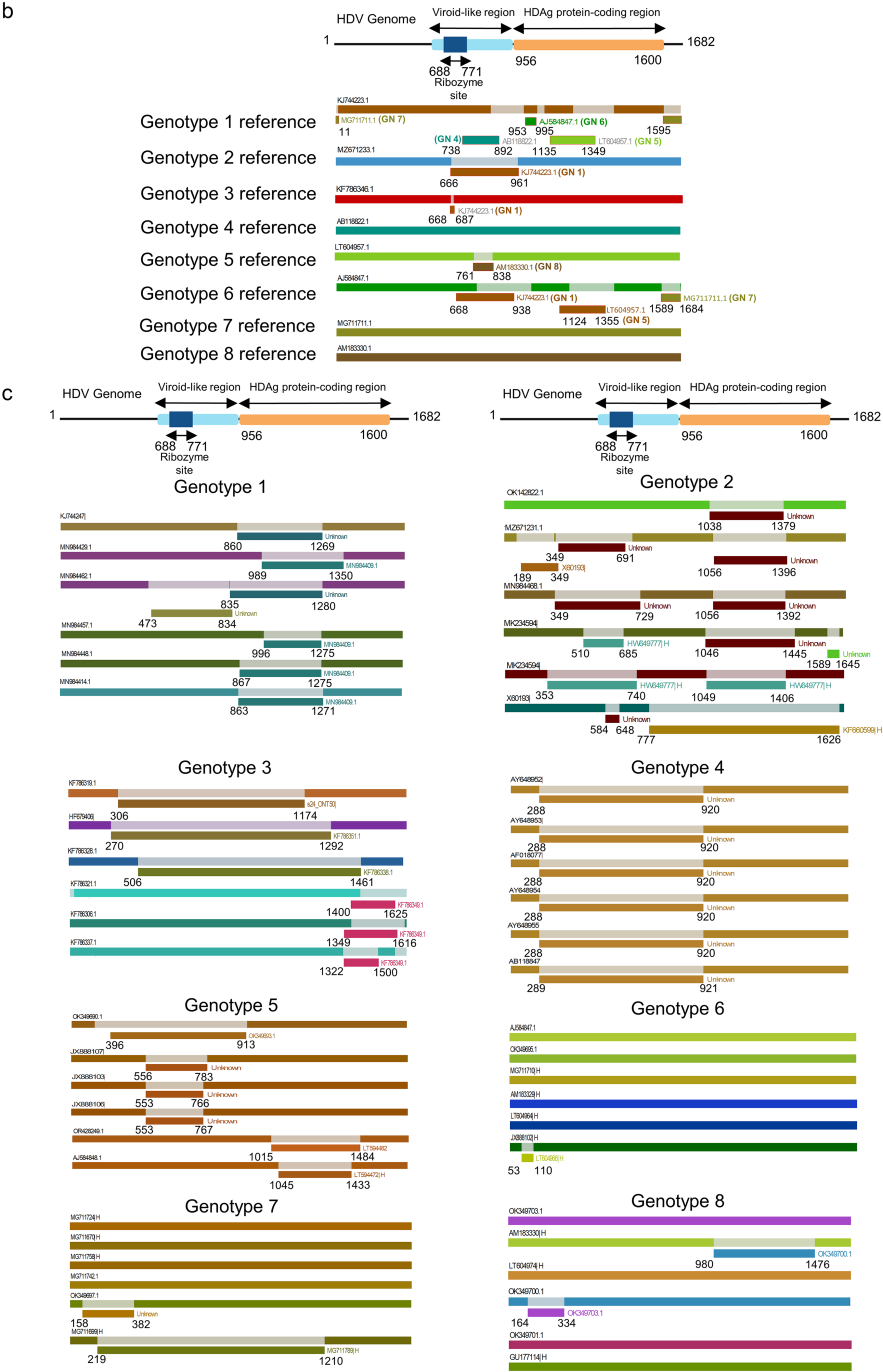
**Recombination across HDV genotypes.** (a, left top) Network tree of 721 complete HDV genome sequences built using SplitsTree4 and published until December 2023. The evolutionary relationship and segregation into the eight HDV genotypes are shown. All published complete HDV genome sequences of the individual genotypes were used to construct the remaining trees. The scale bar of the tree on the top left indicates 0.1 nucleotide substitutions per site, while the remaining scale bars correspond to 0.01 substitutions per site. (b) Recombination analysis carried out on the eight proposed reference sequences using RDP4. (c) Recombination analysis of the individual HDV genotypes. Six representative sequences have been shown exemplarily for each genotype. Recombination events shown have high degrees of confidence with RDP4. Numbers below the sequences indicate the start and end positions of the recombination breakpoints in the genome. Recombination events involving similar sequences are indicated by RDP4 as unknown to avoid misidentification of the parent sequence. Abbreviation: GN, genotype.

The presence of viral recombination in the nucleotide dataset was analysed by constructing recombination network trees. SplitsTree4 is able to compute phylogenetic trees and detect recombination from an alignment of sequences using methods such as split decomposition and neighbour-net analysis. Analysis of 721 complete HDV genome sequences showed the evolutionary relationship and segregation of the HDV genome into eight genotypes and their constituent subgenotypes (Fig. 4a, top left). A single sequence of Genotype 2, KF660598, was placed distal to the Genotype 2 cluster, but close to the Genotype 1 sequences in the tree, indicating that this sequence is evidently more involved in recombination with Genotype 1. Phi tests for recombination on SplitsTree showed no evidence of recombination when all HDV genome sequences were analysed together.

When investigating individual genotypes for their phylogenetic relationships and presence of recombination, recombination was found to be present within the sequences of Genotypes 2 and 4–8, but absent when all 480 sequences of Genotype 1 were analysed together, irrespective of their geographic origin, as well as when analysing sequences of genotype 3 (Fig. 4a). The individual network trees for each genotype also revealed Genotypes 2–5 and 7 splitting into their individual subgenotypes.

As previous studies had shown recombination within Genotype 1 (Bahoussi et al. 2022), its sequences were subsequently grouped by their geographic origin. Indeed, it became evident that recombination was present in the Middle-East Asian and Central Asian sequences of Genotype 1 (Supplementary Fig. S4). Furthermore, significant recombination was detected in six additional cases between different genotypes, including those that were phylogenetically close, such as Genotypes 2 and 5 (Supplementary Fig. S5b) or geographically neighbouring, i.e. Genotypes 2 and 4 or 5 and 7 (Supplementary Fig. S5a, f). Some sequences of Genotypes 2 and 5 were also found to have a higher extent of recombination (Supplementary Fig. S5).

To validate the above-obtained results, we used RDP4 to detect specific recombination sites within the viral genome, visualize sequence breakpoints, and identify parent sequences involved in building the recombinant sequence. Here, events detected by a maximum number of algorithms with a high degree of confidence were chosen as confirmed events of recombination. Upon checking for recombination between the eight proposed reference sequences, significant evidence of recombination was observed between some genotypes. Genotype 1 was found to be involved in recombination with Genotypes 4–7. Additionally, Genotypes 2 and 3 were observed to recombine with Genotype 1, Genotype 5 with Genotype 8, and Genotype 6 with Genotypes 1, 5, and 7 (Fig. 4b). Of note, the recombination sites lay more abundantly in the viroid-like region of the genome, especially along the ribozyme-binding site. Recombination analysis with RDP4 within individual genotypes showed recombination events in all genotypes, with greater evidence of significant events in Genotypes 1–5 (Fig. 4c). Genomic sites such as 288–920 of Genotype 4 and 835–1350 of Genotype 1, the latter including the coding region, were more frequently involved in recombination. RDP4 also found highly significant events of recombination in African (sites 583–1013) and Central Asian (768–1378) sequences of Genotype 1 (Supplementary Figure S6). These sites fell primarily in the ribozyme-harbouring viroid-like region as well as in the L-HDAg protein-coding region of the genome. The combined recombination analyses with SplitsTree and RDP4 found novel recombinant sequences that arose from both intragenotypic and intergenotypic recombination. The sequences that very frequently participated in recombination were MG711691 and MN984409 of Genotype 1; MN984470, HW649777, MZ671231, and KF660599 of Genotype 2; AB118837 of Genotype 4; and MG711699 and MG711789 of Genotype 7.

**Figure 5.**
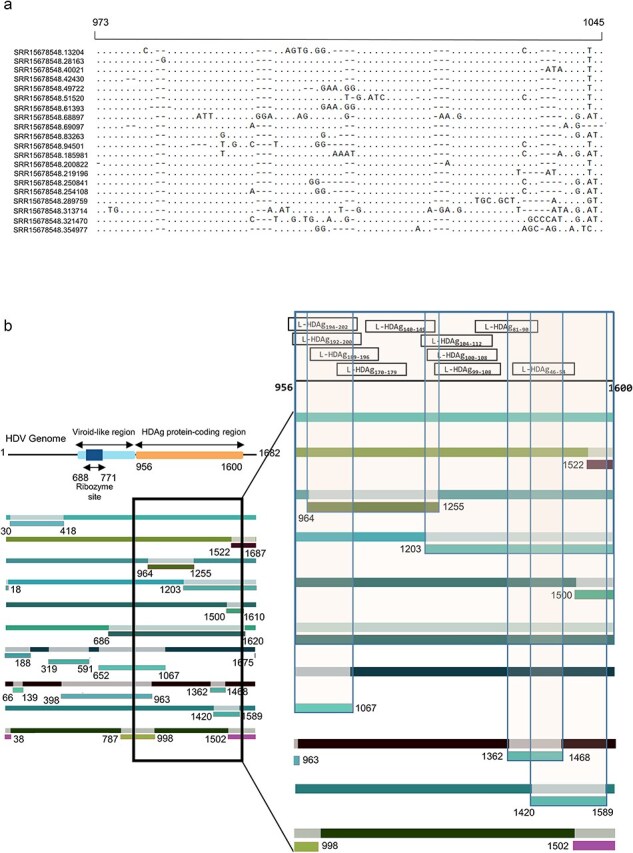
**Long-read sequencing confirms the presence of recombination within HDV sequences.** (a) Multiple sequence alignment conducted on standardized randomly generated full-length HDV sequences from sequencing run SRR15678458. Twenty representative sequences from the hypervariable region of HDV have been shown with their sequence identifiers. (b) Recombination analysis was conducted using RDP4 on the randomly sampled sequences from sequencing run SRR15678458. Ten representative sequences are shown with recombination events displaying high degrees of confidence. The recombination events falling in the CD8^+^ T-cell epitopes of the HDAg-coding site of the genome are highlighted. Numbers denote the breakpoints of the recombinant sequences.

Since most published HDV sequences were generated by Sanger or short-read sequencing, a previously published dataset (BioProject ID PRJNA759204) performed using the Oxford Nanopore long-read sequencing approach was selected to check if recombination was also observed within long-read sequencing data (Charre et al. 2023). Run SRR15678458, corresponding to one HDV-infected patient sample, was chosen for this analysis. A total of 100 full-length genome sequences were randomly sampled out of the 230,000 available sequences. These sequences were further manually standardized to possess the same start and end points as most published sequences. The individual sequences of SRR15678458 generated by long-read sequencing were found to be similar to each other, indicating homogeneity of the sequencing results (Fig. 5a). When applying RDP4 analysis, we found significant levels of recombination of sequences within the same sample. Frequent recombination events were found to span the viroid-like region as well as the variable site of the L-HDAg (Fig. 5b), confirming our previous results and suggesting that recombination may be used by the virus to evade immune recognition.

## Discussion

Previous studies of HDV diversity focused on a limited number of genome sequences or a specific genomic region (Le Gal et al. 2017, Miao et al. 2018, Bahoussi et al. 2022). The current study included all full-length HDV genomes and complete L-HDAg sequences published between 1984 and 2023, providing an unbiased approach to address the conservation and variability of the HDV genome.

So far, most efforts to address questions regarding HDV pathobiology and immunopathology have been conducted by studying Genotype 1 strains, for which a sequence first studied in 1987 was proposed as a reference sequence (NC_001653) (Makino et al. 1987, Saldanha et al. 1990). Aside from that, there are provisional reference sequences for the other HDV genotypes currently available at NCBI, as well as the reference sequences proposed by Miao et al. (2018). Since most of these studies had, however, included a more limited dataset of full-length HDV genomes to determine representative references, we aimed to propose sequences that reflect the currently available data. All reference sequences proposed here have high percentage identities to the consensus sequences created when analysing all available sequences, making them more representative of the available data and thus providing an important tool to address different research questions in future studies.

When analysing the L-HDAg sequence, we could demonstrate that it incorporates regions of high conservation across the different HDV genotypes that could be potentially used for genotype-independent immunotherapeutic strategies. A similar observation of high-level conservation was obtained previously, when comparing 265 L-HDAg sequences across the eight HDV genotypes (Le Gal et al. 2017). Conservation within the L-HDAg suggests that it is important for the viral life cycle, viral stability, and infectivity. Despite the high level of conservation, some of the known epitope regions have amino acid changes affecting *in silico* MHC class I binding, depending on the genotype studied. Since most existing experimental data on HDV-specific T-cell responses have been conducted using Genotype 1-based sequences, as reviewed in Kohsar et al. (2021), the identified epitopes may not be ideal candidates across all genotypes, underscoring the importance of focusing also on non-Genotype 1 viral strains when conducting immunological studies. However, even in the hypervariable C-terminal region of the L-HDAg, most of the amino acid residues across genotypes remain hydrophobic or polar, suggesting that even when variability occurs, the conservation of hydrophobic and polar residues may support the viral life cycle.

Recombination analysis conducted in this study confirms splitting of the HDV sequences into eight genotypes and their subgenotypes, as well as the presence of recombination within all genotypes. This is even more evident when distributing the sequences of Genotype 1 into their corresponding geographical regions of origin, supporting that infections with different strains may lead viruses to recombine. Any noticeable disparity between the results of the tools, SplitsTree4 and RDP4, could be attributed to the algorithms that are used by each tool to detect recombination. While RDP4 found recombination events in all genotypes, SplitsTree4 analysis was unable to detect recombination when all Genotype 1 sequences were analysed together. However, upon distributing the sequences into their associated geographic regions, SplitsTree4 analysis clearly showed evidence of recombination within Genotype 1, indicating that recombination was indeed happening at a geographically local level for these sequences. Furthermore, the analysis showed that recombination is a mechanism of HDV evolution that may additionally affect its recognition by the human immune system. Considerable recombination events are found in the viroid-like regions that harbour the ribozyme-binding site, and this could potentially alter the regulatory control of viral replication or impact HDV’s pathogenicity and fitness. By contrast, recombination events occurring in the HDAg-coding site can alter the protein sequence, and epitope expression, and may thus contribute to evasion from immune recognition. Further studies are required to confirm whether these recombination events are simply a by-product of HDV replication or whether they also enable immune escape. Overall, HDV diversity analyses in the current study provide an important tool to further tailor experimental studies across different genotypes and harmonize efforts to achieve HDV control.

## Supplementary Material

veaf012_Supp

## Data Availability

NCBI GenBank accession numbers of all full-length HDV genomes and L-HDAg sequences incorporated in the nucleotide and protein dataset for the analysis are found in Supplementary Table 1. Raw reads of the previously published Oxford Nanopore long-read sequencing dataset, from which Dr. Caroline Charre provided genomic consensus sequences, can be found under the NCBI BioProject PRJNA759204.
