# Supplementary material for: Sequence analysis of the hepatitis D virus across genotypes reveals highly conserved regions amidst evidence of recombination
Source: Virus Evol. 2025 Feb 27;11(1):veaf012. doi: 10.1093/ve/veaf012 (PMC11927530; doi:10.1093/ve/veaf012)
Supplement: veaf012_Supp [file veaf012_supp.zip › suppl_data/revision_Supplementary_Figures_250225_not_marked.pdf]

Supplementary figure 1

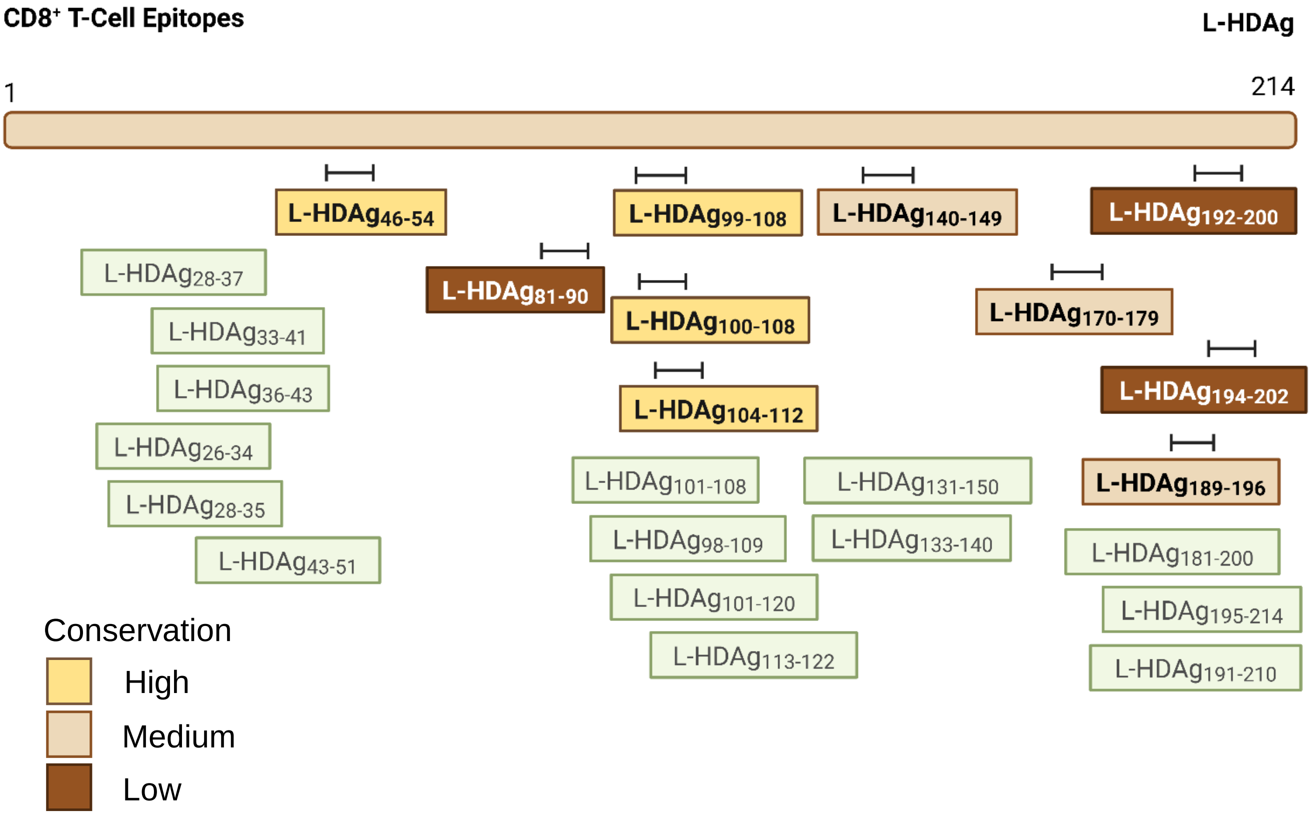


**Supplementary Figure 1: Distribution of confirmed HDV-specific CD8$^+$ T-Cell epitopes across the L-HDAg.** Highlighted in bold are the ten CD8$^+$ T-cell epitopes, confirmed *in vitro*, that have been chosen for our analysis. The thresholds of conservation observed in the epitopes have been based on the total frequency of amino acid positions that remain unchanged across the HDV genotypes – High (yellow): >85%, Medium (beige): 65%-85%, Low (brown): < 65%. The remaining CD8$^+$ T-cell epitopes, including the ones which are currently *in silico* predictions, are depicted in light green. Abbreviations: HDV, hepatitis delta virus; L-HDAg, large hepatitis delta antigen.

# Supplementary figure 2

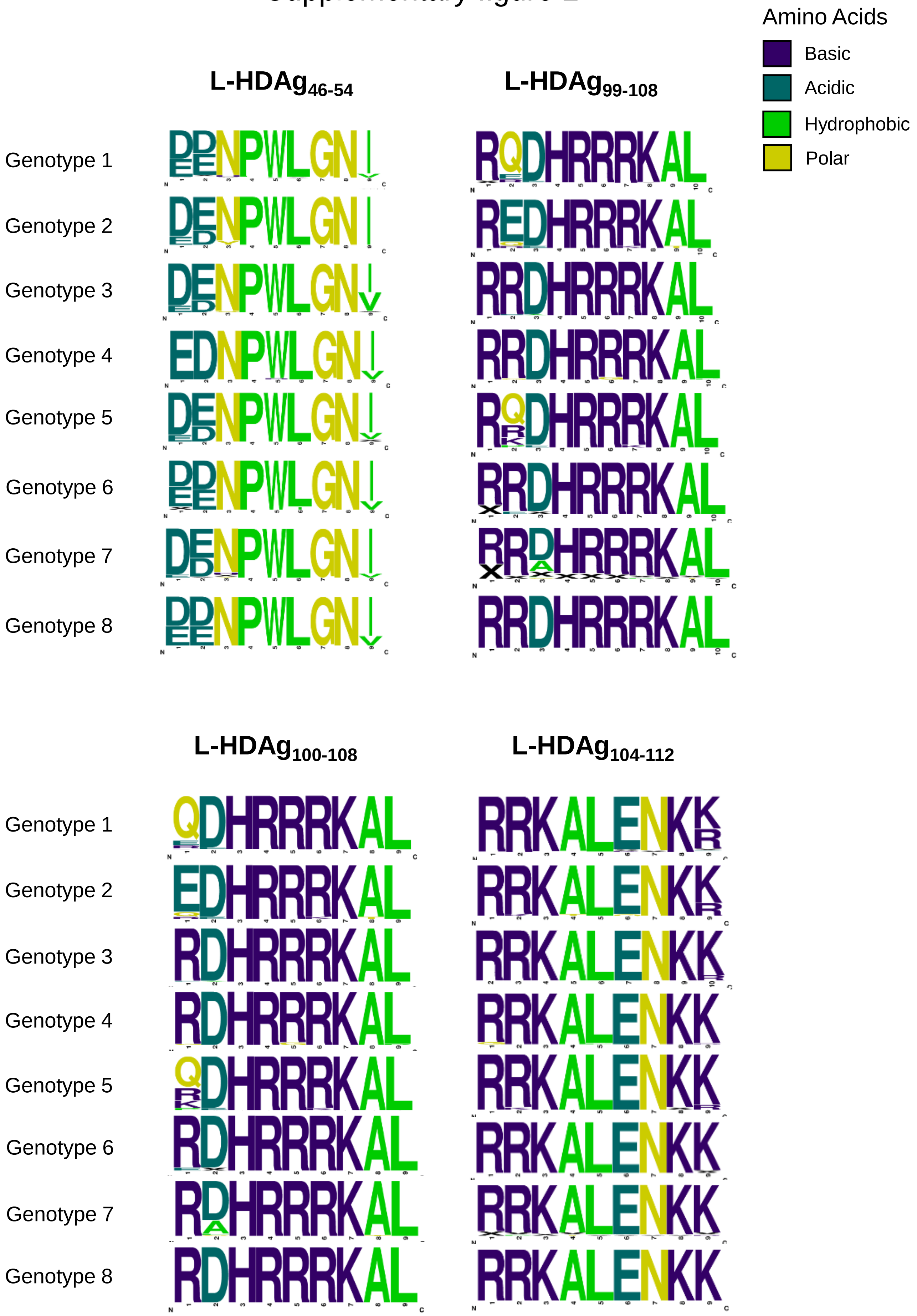

**Supplementary Figure 2: Amino acid variations of L-HDAg sequences within individual HDV genotypes in conserved CD8$^{+}$ T cell epitopes.** Frequency plots visualized using WebLogo show the relative frequency of amino acids for the conserved epitopes in all published L-HDAg sequences of each genotype. Amino acids have been color-coded based on their biochemical properties. Abbreviations: HDV, hepatitis delta virus; L-HDAg, large hepatitis delta antigen.

# Supplementary figure 3

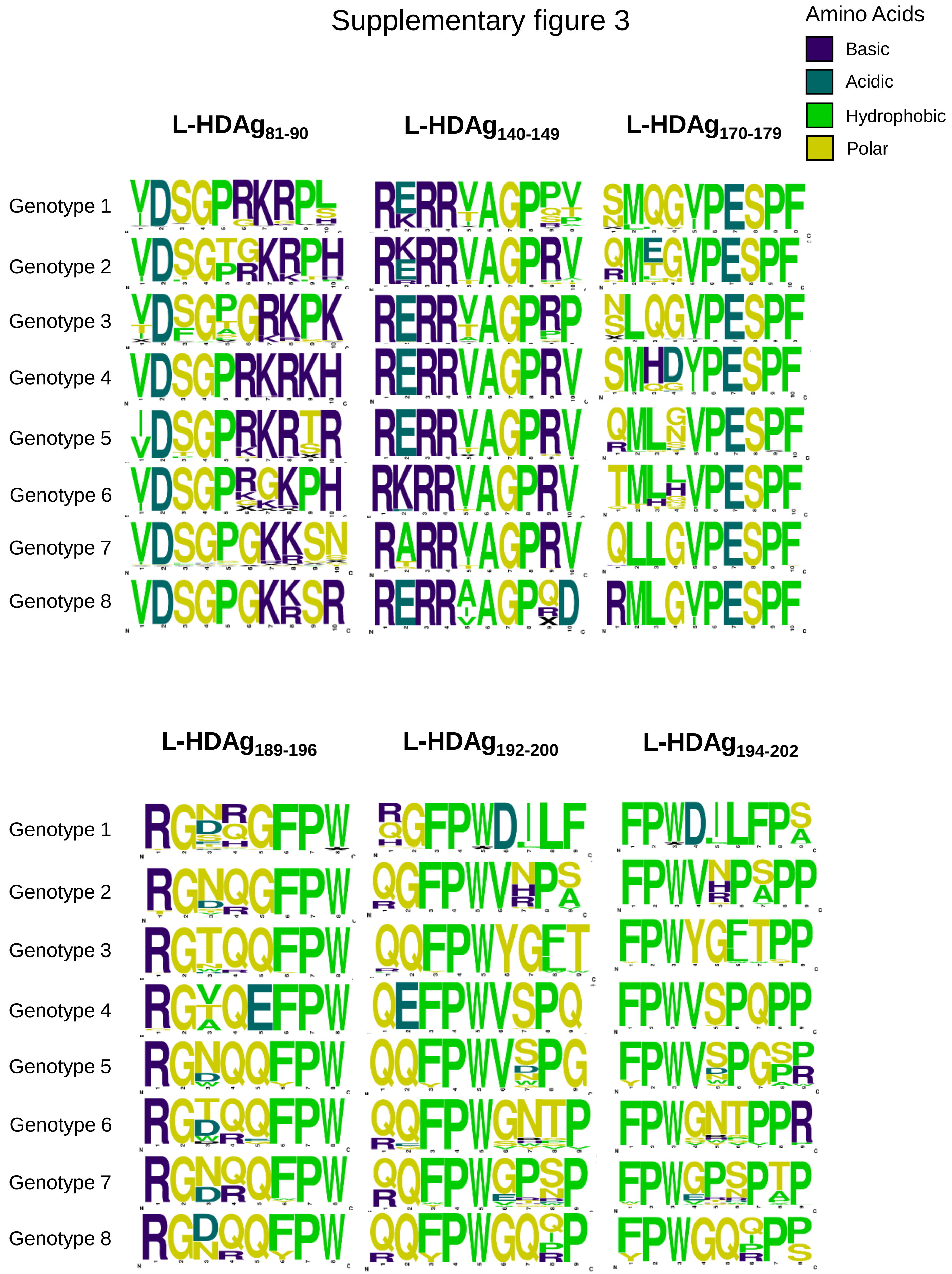

**Supplementary Figure 3: Amino acid variations of L-HDAg sequences within individual HDV genotypes in non-conserved $CD8^+$ T cell epitopes.** Frequency plots visualized using WebLogo show relative frequency of amino acids for the non-conserved epitopes in all published L-HDAg sequences of each genotype. The stop codon site at position 196 was replaced by tryptophan (W) for all L-HDAg sequences. Amino acids have been color-coded based on their biochemical properties. Abbreviations: HDV, hepatitis delta virus; L-HDAg, large hepatitis delta antigen.

# Supplementary figure 4

Genotype 1 – Middle East Asia

Genotype 1 - Central Asia

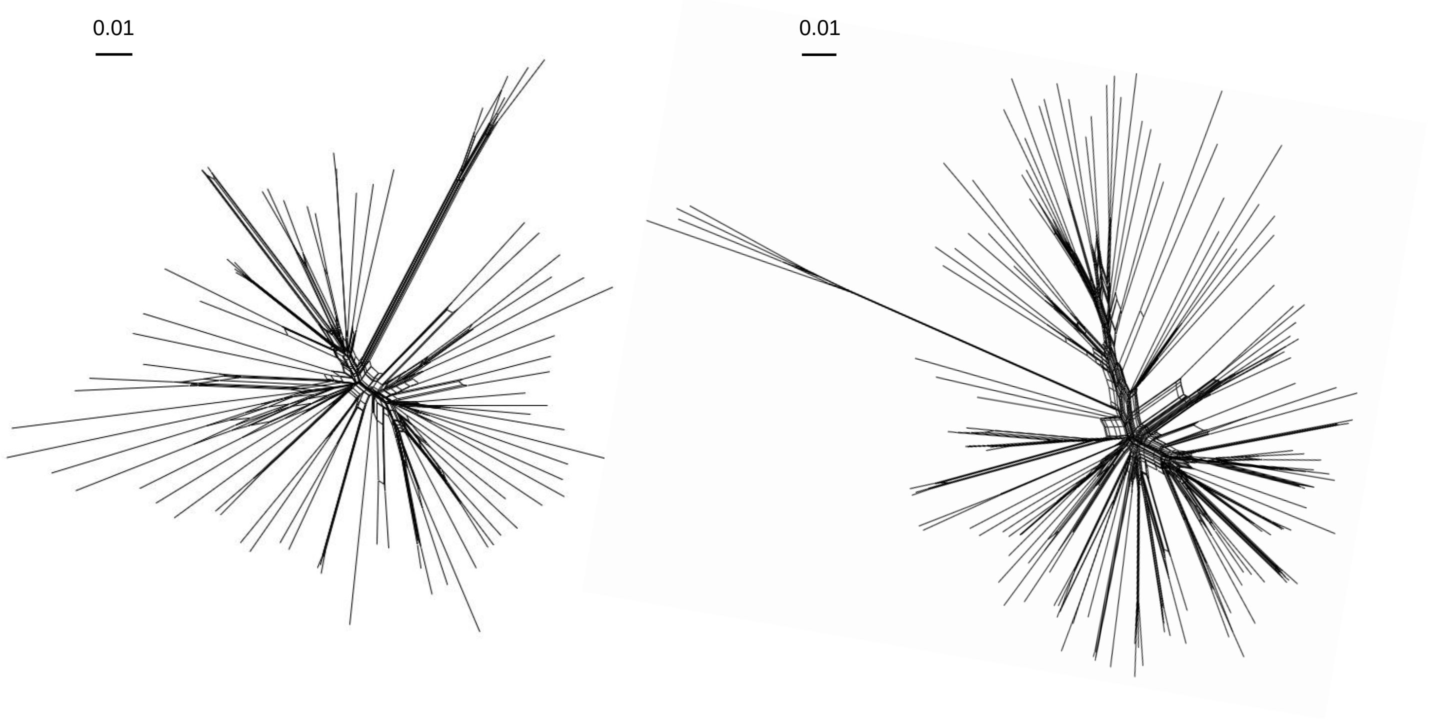


**Supplementary figure 4: Recombination within the different geographic regions of HDV genotype 1 is evident in each SplitsTree network.** 0.01 nucleotide substitutions per site are indicated by the tree scales. Abbreviations: HDV, hepatitis delta virus.

# Supplementary figure 5

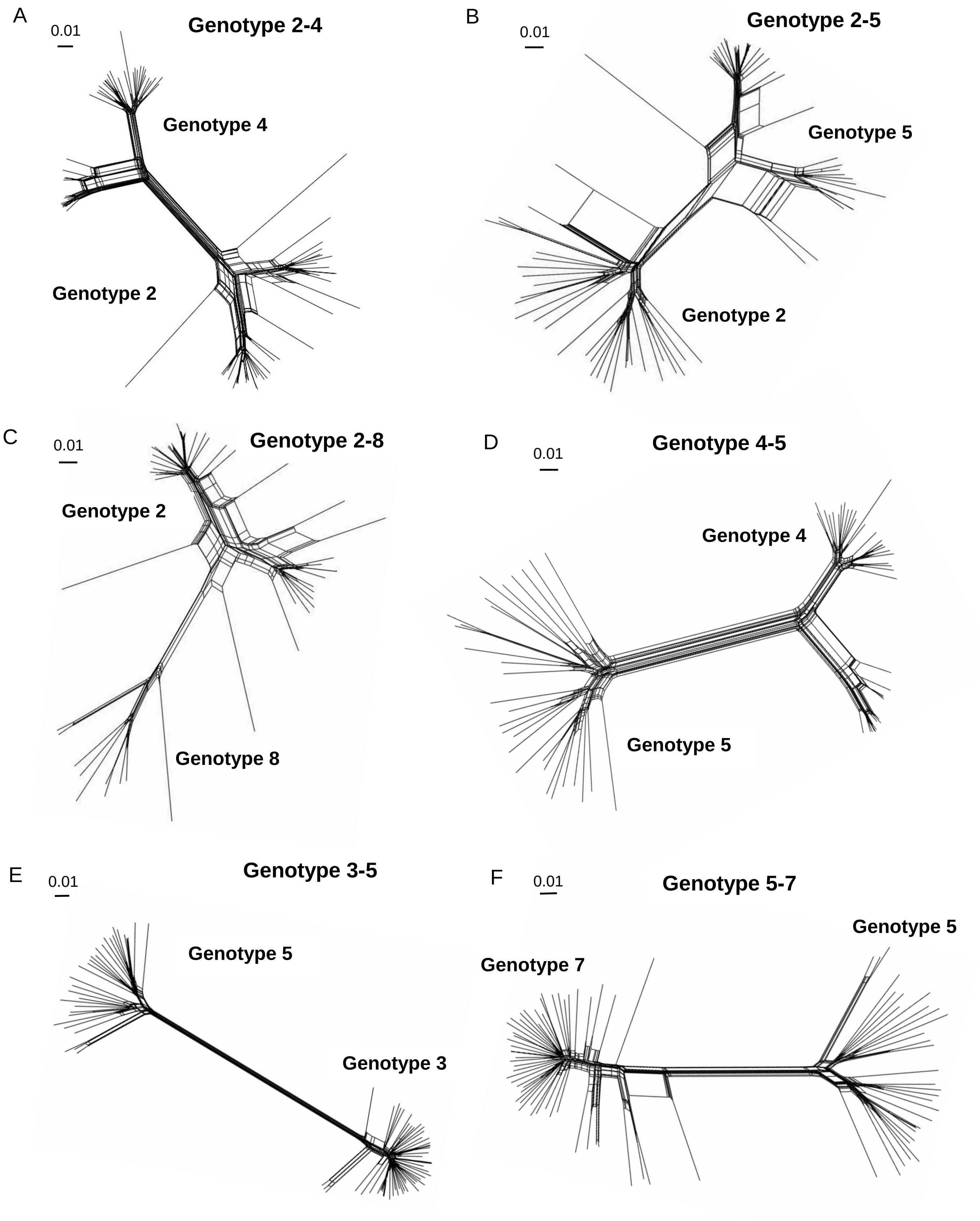


**Supplementary figure 5: Evolutionary relationship between two HDV genotypes.** All possible pairings of two HDV genotypes were analyzed. Six exemplary genotype combinations are shown where recombination is observed between the sequences of two genotypes. The scale length of the trees indicates 0.01 nucleotide substitutions per site. Abbreviations: HDV, hepatitis delta virus.

# Supplementary figure 6

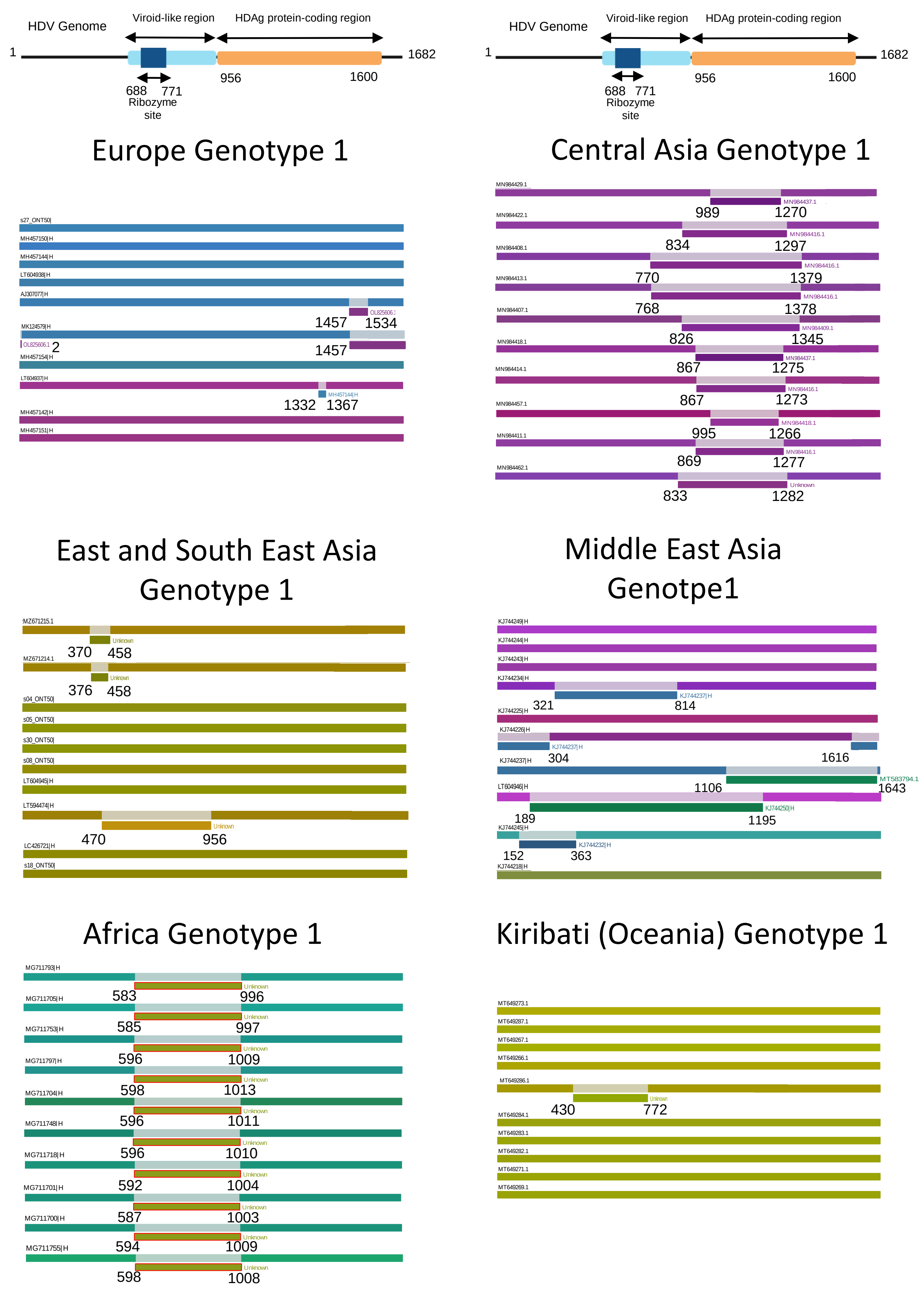

**Supplementary figure 6: Visualization of recombination sites in genotype 1 sequences separated into six different regions of origin.** Recombination events detected using RDP4 with application of GENECONV, MaxChi, RDP, SiScan and Chimaera as recombination-detecting algorithms. The sequences have been compared to the entire length of the HDV genome. Ten representative sequences with recombination events having high degrees of confidence have been shown exemplarily for each region. Numbers below the recombinant sequences indicate the start and end positions of the recombination breakpoints. Recombination events involving similar parent sequences are indicated as unknown by RDP4 to avoid misidentification of the exact sequence. Abbreviations: RDP4, Recombination Detection Program 4; GENECONV, Statistical Tests for Detecting Gene Conversion; MaxChi, Maximum Chi-square; SiScan, sister scanning; HDV, hepatitis delta virus.
