# Supplementary material for: Sequence analysis of the hepatitis D virus across genotypes reveals highly conserved regions amidst evidence of recombination
Source: Virus Evol. 2025 Feb 27;11(1):veaf012. doi: 10.1093/ve/veaf012 (PMC11927530; doi:10.1093/ve/veaf012)
Supplement: veaf012_Supp [file veaf012_supp.zip › suppl_data/revision_Supplementary_Tables_250225_not_marked.pdf]

Table of Contents

Supplementary table 1. NCBI GenBank accession numbers of complete HDV genome and L-HDAg sequences used in the analysis. 2

Supplementary table 2. Altered HDV sequences for standardized reading and analysis. 5

Supplementary table 3. HDV-specific CD8$^+$ T-cell epitopes with their published HLA-restrictions. 6

Supplementary table 4. Consensus sequences of complete HDV genomes per genotype. 7

Supplementary table 5. Percentage identity values and sequence identifiers for the proposed complete genome HDV reference sequences per genotype. 10

Supplementary table 6. Proposed complete genome HDV reference sequences per genotype. 11

Supplementary table 7. Consensus sequences of L-HDAg protein per HDV genotype. 14

Supplementary table 8. Variations in amino acid positions of the L-HDAg within conserved CD8$^+$ T-cell epitopes. 16

Supplementary table 9. Variations in amino acid positions of the L-HDAg within non-conserved CD8$^+$ T-cell epitopes. 19

Supplementary table 1. NCBI GenBank accession numbers of complete HDV genome and L-HDAg sequences used in the analysis.

Genotype	NCBI Accession Numbers	
	Complete HDV Genome	L-HDAg
1	AY633627, AY648956-9, AJ307077, AJ000558, AB118848-9, AF098261, AF425643-4, HW649776, HQ005364-72, HM046802, EF514903-7, L22066, LC426721-2, U81988-9, X04451, OL825606, OK349684-7, OK349704, OQ863057-60, OR428250, KR363257-9, KT722840, KF660600-2, KM110790-801, JX888098-101, JX888104-5, JX888108-13, M21012, M28267, M55042, M84917, MW556198-202, MH457142-54, MH791027-30, MH844625, KY379246-7, KY463677-82, KY495779, KY861350-1, KY861354-5, LT594473-80, LT703299, LT604935-51, LT604968, KJ744214-18, KJ744220-35, KJ744237-8, KJ744240-45, KJ744247-9, KJ744250, KJ744253-7, MT583791, MT583794, MT583796, MT583798, MT583802, MT583804-6, MT583808, MT583812, MT649259-89, MK124579, MK890224-35, MK976661-95, MN984407-67, MN984469, MG926377-82, MG711661-8, MG711672-6, MG711678, MG711681, MG711683-4, MG711686-8, MG711690-4, MG711696-8, MG711712-3, MG711716-8, MG711720-2, MG711725-8, MG711731-4, MG711736-8, MG711740, MG711743-9, MG711750, MG711750, MG711752-3, MG711755-7, MG711759, MG711761-4, MG711766-7, MG711769, MG711770-1, MG711774-6, MG711778, MG711784-5, MG711787-8, MG711792-4, MG711796-8, MG711800-3, MG711805	UBY12330, UBY12332, UBY12334, UBY12338, UBY12340, UBY12342, UBY12344, UBY12346, UBY12348, UBY12350, UBY12352, UBY12354, UBY12356, UBY12358, UBY12360, UBY12362, UBY12364, UGY71091, URH29163, URH29168, URH29170, UXD20269-72, UXD20289, UYO56456, UYO56463, UYO56466, UYO56478, UYO56488, UYO56505-6, UYO56520-1, UYO56535, UYO56538-40, UYO56554-6, SCC98285-301, SCC98318, CAA04189, CAQ16894-936, AAQ09795, AAN17321, AAG26087, AAB59753, AJO72411, AJO72413-4, AJO72418, AJO72424, AJO72430-1, AJO72436, AJO72440, ARB43917, AVO03772-803, AVV65242, AVV65244-5, AYE54588, ABO87233-53, ABP57199-203, AKA64700, AKA64702, AKA64704, AKA64706, AKA64708, AKA64710, AKA64700, AKA64712, AKA64714, AKA64716, AKA64718, AKA64720, AKA64722, ASN65336, ASN65338, ASN65340, ASN65342, ASN65344, ASN65346, AWI66721, AWI66730, AWI66739, AWI66745, AWI66753, AWI66769, AWI6671, AWI66774, ADI24878, ADI24886-8, ALI16950-1, AUG90012-3, AUG90016-7, AXF50889, AXF50894-903, AXF50906-11, AXF50914, AXF50916-7, AXF50919-21,, AXF50923-7, AXF50929-31, AXF50933-9, AXF50942-3, AXF50945-50, AXF50953-56, AXF50959-62, AXF50964-66, AXF50968, AXF50970-77, AXF50979-80, AXF50982-, AXF50986, AXF50988-91, AXF50993-4, AXF50996-1003, AXF51005, AXF51010-11, AXF51013-4, AXF51018-9, AXF51021-2, AXF51024-7, AXF51029, QAX25112, QAX25114, QAX25116, QAX25118, QAX25122, QAX25124, QAX25126, QAX25128, QAX25130, QAX25132, QAX25134, QAX25136, QAX25138, QAX25140, QAX25142, QAX25144, QAX25146, QAX25148, QAX25150, QAX25152, QAX25159, QCC89118, QGQ76699, QGQ76701, QGQ76703, QGQ76705, QGQ76707, QGQ76709, QGQ76711, QGQ76713, QGQ76715, QGQ76717, QGQ76719, QGQ76721, QUE41322-4, QNB92993, QNB92995, QNB92997, QNB92999, QNB93001, QNB93003, QNB93005, QNB93007, QNB93009, QNB93011, QNB93013, QNB93015, QNB93017, QNB93019, QNB93021, QNB93023, QNB93025, QNB93027, QNB93029, QNB93031, QNB93033, QNB93035, QNB93037, QNB93039, QNB93041, QNB93043, QNB93045, QNB93047, QNB93049, QNB93051, QNB93053, QND75904-5, QND75913, QND75915, QND75917, QNQ17903, QNQ17905, QNQ17907, QNQ17909, QNQ17911, QNQ17913, QNQ17915, QNQ17917, QNQ17919, QNQ17921, QNQ17923, QNQ17925, QNQ17927, QNQ17929, QNQ17931, QNQ17933, QNQ17935, QNQ17937, QNQ1790339, QNQ17941, QNQ17943, QNQ17945, QNQ17947, QNQ17949, QNQ17951, QNQ17953, QNQ17955, QNQ17957, QNQ17959, QNQ17961, QNQ17963, QNQ17965, QNQ17967, QNQ17969, QNQ17971, QNQ17973, QNQ17975, QNQ17977, QNQ17979, QNQ17981, QNQ17983, QNQ17985, QNQ17987, QNQ17989, QNQ17991, QNQ17993, QNQ17995, QNQ17997, QNQ17999, QNQ18001, QNQ18003, QNQ18005, QNQ18007, QNQ18009, QNQ18011, QNQ18013, QNQ18015, QNQ18017, QNQ18019, QNQ18023, WGU42319, WGH70920, WGH70922, WGH70924, WGH70926, WGH70928, WGH70930, WGH70932, WGH70934, WGH70936, WGH70938,

		WGH70940, WGH70942, WGH70944, WGH70946, WGH70948, WGH70950, WGH70952, WGH70954, WGH70956, WGH70958, WGH70960, WGH70962, WGH70964, WGH70966, WGH70968, WGH70970, WGH70972, WGH70974, WGH70976, WGH70978, WGH70980, WGH70982, WGH70984, WGH70986, WGH70988, WGH70990, WGH70992, WGH70994, WGH70996, WGH70998, WGH71000, WGH71002, WGH71004, WGH71006, WGH71008, WGH71010, WGH71012, WGH71014, WGH71016, WGH71018, WGH71020, WGH71022, WGH71024, WGH71026, WGH71028, WGH71030, WGH71032, WGH71034, WGH71036, WGH71038, WGH71040, WGH71042, WGH71044, WGH71046, WGH71048, WGH71050, WGH71052, WGH71054, WGH71056, WGH71058, WGH71060, WGH71062, WGH71064, WGH71066, WGH71068, WGH71070, WGH71072, WGH71074, WGH71076, WGH71078, WGH71080, WGH71082, WGH71084, WGH71086, WGH71088, WGH71090, WGH71092, WGH71094, WGH710996, WGH71098, WGH71100, WGH71102, WGH71104, WGH71106, WGH71108, WGH71110, WGH71112, WGH71114, WGH71116, WGH71118, WGH71120, WGH71122, WGH71124, WGH71126, WGH71128, WGH71130, WGH71132, WGH71134, WGH71136, WGH71138, WGH71140, WGH71142, WGH71144, WGH71146, WGH71148, WGH71150, WGH71152, WGH71154, WGH71156, WGH71158, WGH71160, WGH71161, WGH71162, WGH71164, WGH71166, WGH71168, WGH71170, WGH71172, WGH71174, WGH71176, WGH71178, WGH71180, WGH71182
2	OK142820, OK142822-3, OK1428326, OK142828, OK1428330, OK142832, OK1428355, U19598, MN98446, MN984470, MZ671229-33, AB118846, MK234591-4, LT604952-3, LT604981, MG557658-9, AY261457-60, X60193, AJ309879-80, MK234591-4, HW649777, AF104245, AF104264, KF660598-9	AAG26088, AXT99879-80, SCC98302, AAC55090, QNQ18021, ABO87254-57, UYO56437, UYO56439, UYO56440, UYO56443, UYO56445, UYO56447, UYO56449, UYO56472, UBY12366, UBY12368, UBY12370, UBY12372, UBY12374
3	L22063, LT604954-5, KC590319, HW649778, HF679404-6, AB037947-9, KF786305- KF786306, KF786308-11, KF786313-15, KF786317-19, KF786321, KF786323-40, KF786342-44, KF786346-52	SCC98304-5, AIR77005-6, AIR77008-11, AIR77013-15, AIR77017, AIR77021, AIR77023-43, AIR77046-51
4	MN401236, MT050453, AY648952-5, AF309420, AF018077, AF209859, AB088679, AB118819-45, AB118847	ABO87258- ABO87296
5	AJ584845-6, AJ584848, OK349688-93, OR428249, AM183326, AM183328, AM183331, JX888103, JX888106-7, LT594456-63, LT594472, LT594482-3	CAE51160-61, CAE51163, UXD20273-78, CAJ66090, CAJ66095, SCC98306-13

6	OK349694-6, AJ584847, JX888102, KY861353, AM183329, AM183332, LT604964-7, LT604984-5, MG711671, MG711710, MG711730, MG711777, MG711780	UXD20279-81, AUG90015, CAJ66093, CAJ66096, CAE51162, SCC98303, SCC98314-17, AXF50905, AXF50940, AXF50958, AXF51004, AXF51007
7	OK349697-9, KM110802-5, LT594442, LT594469-72, LT594486-7, AM183333, MG711670, MG711679, MG711680, MG711682, MG711685, MG711689, MG711695, MG711699, MG711711, MG7116714, MG711723-4, MG711729, MG711735, MG711739, MG711741-2, MG711751, MG711754, MG711758, MG711760, MG711765, MG711768, MG711772, MG711773, MG711779, MG711781, MG711783, MG711786, MG711789, MG711790-1, MG711795, MG711799, MG711804	CAJ66097, UXD20282-84, AKA64724, AKA64726, AKA64728, AKA64730, SCC98319-22, AXF50892, AXF50904, AXF50912-13, AXF50915, AXF50918, AXF50922, AXF50928, AXF50932, AXF50941, AXF50944, AXF50951-52, AXF50957, AXF50963, AXF50967, AXF50969, AXF50978, AXF50981, AXF50985, AXF50987, AXF50992, AXF50995, AXF50999-1000, AXF51006, AXF51008, AXF51009, AXF51012, AXF51015-1017, AXF51020, AXF51023, AXF51028
8	OK349700-3, LT604973-4, LT594488, GU177114, AM183327, AM183330	CAJ66091, SCC98323-24, UXD20286.1, UXD20288.1

Abbreviations: NCBI, National Center for Biotechnology Information; HDV, hepatitis delta virus; L-HDAg, large hepatitis delta antigen.

Supplementary table 2. Altered HDV sequences for standardized reading and analysis.

Genotype	NCBI Accession Number	Alteration
1	HQ005364-72, KY861350- KY861351, KY861354- KY861355, MG711661- MG711662, MG711664- MG711667, MG711672- MG711676, MG711678, MG711681, MG711683- MG711684, MG711686- MG711688, MG711690- MG711694, MG711697- MG711698, MG711700- MG711701, MG711703- MG711706, MG711709, MG711713, MG711716, MG711718, MG711720- MG711722, MG711725- MG711728, MG711731- MG711734, MG711736- MG711738, MG711744- MG711746, MG711748- MG711749, MG711750, MG711752- MG711753, MG711755- MG711757, MG711759, MG711761- MG711764, MG711766- MG711767, MG711769, MG711770- MG711771, MG711774- MG711776, MG711778, MG711784- MG711785, MG711787- MG711788, MG711792- MG711794, MG711796- MG711798, MG711801, MG711803, MG711805	Altered to read from standardized initial reading site
3	KF786305- KF786306, KF786308- KF786311, KF786313- KF786315, KF786317- KF786319, KF786321, KF786323- KF786340, KF786342- KF786344, KF786346- KF786352	Antigenome converted to genomic sequences
6	KY861353, MG711671, MG711710, MG711730, MG711777, MG711780	Altered to read from standardized initial reading site
7	MG711679, MG711680, MG711685, , MG711689, MG711699, MG711711, MG711714, MG711723, MG711724, MG711729, MG711735, MG711739, MG711741, MG711751, MG711758, MG711760, MG711765, MG711768, MG711772- MG711773, MG711779- MG711781, MG711783, MG711786, MG711789- MG711791, MG711799, MG711804	Altered to read from standardized initial reading site

Abbreviations: HDV, hepatitis delta virus; NCBI, National Center for Biotechnology Information

Supplementary table 3. HDV-specific CD8^{+} T-cell epitopes with their published HLA-restrictions.

CD8^{+} T-cell Epitope	Sequence	HLA-restriction
**L-HDAg$_{46-54}$**	**DENPWLGNI**	**B*18:01**
**L-HDAg$_{81-90}$**	**VDSGPRKRPL**	**B*37:01**
**L-HDAg$_{99-108}$**	**RRDHRRRKAL**	**B*27:05**
**L-HDAg$_{100-108}$**	**QDHRRRKAL**	**B*37:01**
**L-HDAg$_{104-112}$**	**RRKALENK/R**	**B*27:05**
**L-HDAg$_{140-149}$**	**RERRVAGPPV**	**B*41:01**
**L-HDAg$_{170-179}$**	**SMQGVPESPF**	**B*15:01**
**L-HDAg$_{189-196}$**	**RGSQGFPW**	**B*58:01**
**L-HDAg$_{192-200}$**	**QGFPWDILF**	**B*35:01**
**L-HDAg$_{194-202}$**	**FPWDILFPA**	**B*35:01; B*07:02**
L-HDAg$_{98-109}$	ERRDHRRRKALE	B*27:05
L-HDAg$_{26-34}$	KLEDLERDL	A*02:01
L-HDAg$_{43-51}$	KLEDENPWL	A*02:01
L-HDAg$_{191-210}$	GQGFPWDILFPS	B*35:01; B*51:01; B*53:01
L-HDAg$_{101-120}$	DHRRRKALENKR	A*03:01
L-HDAg$_{131-150}$	KRLTEEDERRER	A*02:02P/03:01P; B* 15:01P/41:01; C*03:04/17:01P
L-HDAg$_{181-200}$	RHGEGLGVRGG	B*15:01; C*04:01
L-HDAg$_{195-214}$	PWDILFPSDPPF	A*02:17/02:01; B*35:01
L-HDAg$_{33–41}$	DLRKVKKKI	B*13
L-HDAg$_{36-43}$	KVKKKIKK	A*30
L-HDAg$_{28–37}$	EELERDLRKV	B*49
L-HDAg$_{28–35}$	EELERDLR	A*33
L-HDAg$_{101–108}$	DHRRRKAL	B*14
L-HDAg$_{113–122}$	KQLSAGGKNL	B*13
L-HDAg$_{133–140}$	LTEEDERR	A*68

Highlighted in bold are the ten CD8^{+} T-cell epitopes with their confirmed HLA-restrictions that were used for this analysis. Abbreviations: L-HDAg, large hepatitis delta antigen; HLA, Human Leukocyte Antigen.

Supplementary table 4. Consensus sequences of complete HDV genomes per genotype.

Genotype	Consensus sequences of Complete HDV Genomes
1	-------------------ATG------AG-CCA-------------------------------------NNGTTCCG--AN-CGAAGNG-NNCGC---G--GNGGGNA-GG-A-TCAGCNCCC-GAGA-GGG---<br>--------------------------GNTN-NNN-GGTAAAG-AG--C-NNNGGAA-C-GTCGGN--GG-NA--NNNCTCCCAAG-AAG--NNN-AAAA-G-AGA---A--NNCAAGAAN----<br>CGGAN-G-NTTTCCC-CAT--GACGCTGG--AGA-CA--TCTT-GGAA-----GGGGAAA--G--ANGGAAGGTGGAA-AAG-AAA-GGGG--C-GGG-CCTC-CC--<br>GATCC----GAGGGG------CCCGAC-----NNCCA-A--G-TTT--GGAGAGC-AC--TC--CGGGCCG-----A--ANGGTT--GAGTA-GCA-C-CCAGA-GGG-AG-G-A-A-<br>TCCACAC---------GGAG-AANAGCAGAN--AA-------A----------TC-ACCT-CCAG-AGG----A-CCCCT---TCAGCGAAC-AG-AA-GA-GCNNNNCNC-GCGNN---<br>AGG------AGTAAGACCATAGCGAT-AGGGGG--AGATGCTAGGAG-TTAGN------------GGAGACCGA--AGC---GA-GG-A-GG-AAAGC-AAAGAAAGCAACG<br>GGGCTAGCCGGTGGGTGTTCCGCCCCCCGAGAGGGGACGAGTGAGGCTTATCCCGGGGAACTCG-----GCGAA-TCGTCCCCACATAGC-AGN-NCC-<br>CCGGACCCCCTTC--C-AAAATGA--CCGAG-GGGGG-NNGCTA-GGAGCG--C--GGGGG---A---CCN-G-T-GGA-----G-CCATGG-GA---TGC-T-CT-T-CCC<br>GAT--TCCG---TCC--A-T--C----CCCN--CCCCC-N----GAG-GGT-CG-CCC-AG---GAATGGC--GG--G------A-CCCCA--CTCN-NN-C-AGGGTCCGC-GTTCCA<br>T-CC-TTTCTTACCTGATGGCC-G-GCATGGTCCCAGCCT-CCTCGCTGGCGCCGGCTGGGCAACATTCCG-AGGGGACCGT-CC-CCTCGGTAATGGC<br>GAAT--GGGA-CCCAGAAA--TCTCTCTA-GATTCCGATAGA------GAATC--GAGA-G----AAAA-CTGGCTCT-CCCTTAGCCATCCG-A-G--T--GGACGTNN<br>GTCC-TCCTTCGGAT-GCCCAGGTCGGACCGCGA------GGAG-GTGGAGATGCCAT-GCCGA-CCCGAAG--AGGAAAGAAGGACGCGAGAC-GCGAA-<br>CCTGTGAGTGG-AAACCCG-CTTTATTCA---CTGGGGTCGACAACTCTGGGGAGAAAAGGGAGGATCGGATGGGAAGAGTA-TATCCTACGGGAAT<br>CCCNGGTTTCCCC---TCANGTCCAGCCCCTCCCCGGTCCTGGNGAAGGGGGACTCCGGGACTCCTTGCATGCT-GGGGACGAAGCCGCCCCCGGG<br>CGCTCCCC--TCGA-----TCCACCTTCGAGGGGGTTCACACCCCCAAC---CGGCGGGCCGGCTACTCTTCTTTCCCTTCTCTCGTCTTCCTCGGTCAACC<br>TCTTAAGTTCCTCTTCTTCCTCCTTGCTGAGGTTCTTCCCT---CCCGCGGNNAGCTGCTTCTTCTTGTTCTCGAGGGCC<br>TTCC---------------TTCGTCGGTG-----------------------------------A------TCCTGC------CTCTCCTTGTCGGTGAATCCTCCCCTGAGAGGCCTCTTCCTAGG<br>TCCGGAGTCTACCTCCA-TCTGGTCCGTTCGGGC---CC---TCTTCGCC---GGGGGA---GCCCCCTC-TCCATCCTTATCCTTCTTTCCGAGAATTCCTTTG<br>ATGTTTCCCAGCCAGGGATTNTCNTCCTCNAGTTTCTTGA-------------------TTTTC--TTCTTNACCTTCCGGAGNT-CCCTCTCGAGTTCCTCTAACTTC<br>TTTCTTCCANTCACCCACTGCTCGAGGATCTCTTCTC-TTCCCCCGCGG---TTCTTCTTCGANTCGGACCGGCTCATC-T-CGACAAGAGGC-G—GCNG<br>TCCTCAGTGCTCTT-ACTCTTT-TC-T--G-T--AAAGAGGAGA-CTGCTGGA--CTCGCCG-CCCAAGTTCGAG--------
2	-ATGGGCCGCA-GCCGGGCGANGGGGGCGAAGT-GGGGAGGGGGAGATCCCGAGGNGGATCNCCAACTAAAGAGTGGAGGAAATCTCGG<br>AAAGCTTCTCCCAAGAAGAACCGGGAN-GCTCTCAAGAGGGAGGA--GATNTCCCGAACGCTG---GAGAC-T-CCCGGAATNTCANTCAAGAAGAAGGT<br>AACGGAGAAAGGAGCGGGCCTCCCGATCCGAGTGGTCCGCG-GCGTATCGAGTTTGGAG-TCATCCGGN-CGAAGGGTTGAGATG-ACCCACAGTC<br>GGGTGA-TCCACCAGGAGGG-TGGAGGAGNATTCACCTCCAGAGGACCCCTTC-AGCGAACAGAAGAGCC------TCCTCCTNCGGAGAGA----AAGACC<br>ATAGCGATGGGGGGAGATGCTAGGAGTNGGNGGCGACCGAAGCGAGGNGG-AAAGNAAAGAAAGCAACGGGGCTAGCGAGTGGATGTTCCGCC-C<br>CCCGGCGGGGCCGAGTGAGGCTTATCCCGGGGA--TCGGCGAATCGTCCCGGGATGGGGACTCC--NAGAGCCCCTTCCGAAGNGACGGGG-<br>GGGGGTCACTAGGAACACNGGGGA--CCAGTGGATCCGTGGG-ATTA-CCCTCTCCACCCTCCCAACACACTCCTTCCCCCCTGCGGGGCCCCCCC<br>ATAAGATGGCAGGAACCCACTCATTGGGGTCCGCTGT-TCCATTCTTTCTTACCTTGTGGCCGGCATGGTCCCAGCCT-CCTCGCTGGCGCCGGCT<br>GGGCAACATTCCG-AGGGGACCGTCCCTCGGTAATGGCGAATGGGACCCAGAACTCTCTCTAGATTCCCAGAGAGAATCGAGAGAAAACTGGCTCT<br>CCCTTAGCCATCCGAGT-GGACGTTCGTCCTCCTACGGATGCCCAGGTCGGACCGCGGGGAGGTGGAGATGCCATGCCGACCCGAAGAGGAAA<br>GAAGAACTCGGACGCGAA-CCGTGAGTGGAACCCTNGTTCTTTATTGGGGGGTACACTCGAGGAGTGGAAGGCGTTGTT—GGGGGGGAGATGGG<br>TNTACCTACGGGAATCCCTGGTTTCCTCTGATGTCCAGTCCCTCCCCCGTCCGAGNGAAGGGNGACTCCGGAACTCCNTCCATCTGAGGGACGAAGC<br>CGCCACCGGGCGCTCCCCTCGGACCTCCTCCGGGGGGGTTCACATCCCCAACCCGCGGGCCGGCTACTCTTCTTTTCCTTTCCTCGTCTTCAACGGT<br>CAACCTCCNGAGTTCCTCTTCTTCCTCCCTGCTGAGGCTCTTTCCTCCCGAGGAGAGCTGCTTCTTCTTGTTCTCGAGGGCCTTCCTTCTGCGGTGAT<br>CCTCCCTCTCCTTGTCGGTGAACCCGCTCTTGTGAGGCCTCTTCCCAGTCCCGGAGTCGACCTCCATCTGATCCGTCCGGGNCCTCTTCGCCGGGGG<br>AGCCCCCTCCCCGTCCTT-CCCTTTTCTGATTATTCCGAGGATGTTCCCCAGCCAGGGATTTTCATCCTCGAGTCTCTTGATGGTCTTCCTG<br>GCCTTCCGGAGGTCTTTCTCGAGCTCCTCCGCCTTTCTTCTTGTTGTGATCCATTTCTCGAGGATNTCCTCCCTTNNTCCCCTTCGNCTTCTCCTCG<br>ACTCGGATTGGCTCATCCTCGGNNCGAGGCGANGGT--CCTCAGTTCTCTCTATTCTTTCCTT-TTG-AAAGAGGAGACTGCTGGAC-CAAACGCCCGA<br>GCCGGG---
3	---TGGGCCGCTT-CCGGCGAAGGGGCCCGAAG-TNGNGAGNGGNGGAGAAC-TCCCGAGAGTTGGGAGACAGAAAGAGGGCGAATGGCCTCGGAT<br>GG-ATCCCCCAAGCTGCCAACAGAGGAAGAAAACAAGANAGAGGAGCCCCCATGACGCTGGCGAACCATCGCGNACTGGGAGAGGAGTGGCGAA<br>GA-NA--GN-AGCGGAGACCCC-GGTCCGAATGCCCATCGGCAGCAAACTCCTCTGGAGTCCTCCGGANCC-AAAGGAGA-AAACTACCGGCGGAGGG<br>TGATCCACCCGGAGNTGGACGGACAAG-CCACATCCAGAGGACCCCTTCGNCGAACAGAAGACCCTGGTACCGGGGGGGAATAGCCCATAGTACAA<br>GGGGAGATGCTAGGAGTCGGGGGAAGCCAGAACGACTGGGAAAG-AAAAGAAAGCAACGGGGCTAGCTGCCGGGTGTTCCATCCATGGGATC-GGT<br>GCCGAGTGAGGCTTATCCC-GGGGGTGACGCCTCGGTCCTC-CCTTAGCATCGGAGT-CCCGGGCCCCCTCCCAGGAATGGGAACAGGGGGAGATC<br>GACCGGG-TCCCGCAGAACCCGATGGAG--TNCCCCACC-ATCCCTTNCCGGANA-AAACTGGTCCCGATAGGGGC-ACCCACAATAGGATGGCAANA<br>GGGGACTCTCGGGTCCGTCGTTCCATCCTTTTCTTACCTTGT-GGCCGGCATGGCCCCAGCCTCCTCGCTGGCGCCGGCTGGGCAACGATCCGAG<br>GGAACTATTCCTCTCGAGAATCGGCAAATGGGG-CCCCTCGCTCGTATCTCCGAGAGGAGACGAGAAGGAGGTGGATCTCCCTTTGCCATCCGAGG<br>GAACT-ACGTTCTCCTTACGGATGCCCAGGTCGGACCGCGAGGAGGTGGAGATCCCATGCCGACCCGAAG-AGGAAAGGAGGACACGGACGACAAA<br>CCGTGAGTTCTATTGCCCTTTATTGTTGGGTGCACCCTGGGACCCAGTAATACCCGGGGGGAGGCGGGGTAAACCCATA-C-ATGGGAACTGCTGGG<br>TTCCTCGGATGTCGATCCCCTCTCCCGTTCGGGAAAAGGGGGACTCCGGAACTCCCTGCAGGCTGGGCACGAAGCCCCCACCGGGCGCTCCCCTCG<br>GCGGGCCGTCCATTGGGTTCACACCCCCAGGCCGCGGGCCGGCTACTCTTCTTTCTCTTTCGTCGTCATCCNTGGCCAGCCTCCGGAGTTCCTCTTC<br>TTCCTCTTGGCTGAGGTGCTTTCCCCCTCCGGCCAGTTGCTTCTTCTTGTTCTCCAGGGCCTTC---CTTCTTCTGTGGTCCCGCCTCTCCTTGT----CGG-<br>-----TGAA--CCCCCTGGCCTTGGGTTTCCTCCCAGGTCCGGAATCAACCTCCATGGTTTCCTGCCTGGGCCTCTTCGCCGGGGGCCCTCCGT<br>CTTCGTCCTTCTTCCT---TCTCAACAGTCCGACGATGTTTCCCAGCCAGGGGTTTTCATCCTCAAGTTTCTTGATCTTCTTGTTGGTTCGCCGGAGAT<br>CCTTCTCGAGTATTCGCCTTTTCTTCCTTTCTTCTACCCACTGTTCGAGGATCTCCTCCCTCTCCTTCGAGGNCGGCTTCGCGACGGNTTGGCTCATCC<br>TGAGACCGGGGAGCTTCGACGATCTCTT-TNATCTCCTGAAG-GAGGAAGGAGCTCTCGAACGCCCCCCCGNCTCCTCGGA—

**4**	GATGGGCCNCAG-TGCCGACGAGGAGTCCGCAGGTGGGAGGATCAGCCACCGGAGAGGGACGCGATGGC-AAGAGTGGAGGAAAGTTCG-GAGGG<br>AAATCCCAAGAAGGTCANTCGAGATTCAAGNGGTGA-GGAGGGATCCCCGAGACGCTGGAGGAGCCCCGGGAAAAAANN-AAAAAAGGCAAGGGAT<br>TGGTAGAAAAGAGCG-AGCCTCCCGA-TACGAGTTTGCCAGGACCTATC-AAGTTTGGAGTCATCCGGGCCGTAGGGGAGAATAGAACACCGGGGG<br>GTGATCCACCAGGAGAAGNAGCGGAGAACCCACCTCCAGAGGACCCCTTCAGCGAACAGAAAAGCTCCTCCTCNNCGGGAGTAGGNCCGTAGCGAT<br>GGGGGGAGATGCTAGGAGTTGGAGGAGACCGAAGCGAGGAGGAAAGCAAAGAAAGCAACGGGGCTAGCGAGTGGATGTTCCTCCCCCCGAG-<br>GGTCCCGAGTGAGGCTTATCCCGGGGATCGGCCTCCGCCCCNCC--ATGGNGGACTCCAGGACCCCCN-NAAAGGAGGGGGGTGGGCCTTGGACC<br>CTCAGGGAG--CCATGGGATCCGTGGAGTACCC-GGGCCATCCCCTTCTCCACACTCCTTCCCCCCT-GCGGGG-CCCCCCCATAAGATGGCGAGAA<br>ATCCACTCA-TGGGTCCNTCGTCCATCTC—TTTCTTACCTTTTGGCCGGCATGGTCCCAGCCTCCTCGTTGGCGCCGGCCGGGCAACATTCCGAAG<br>GGGACCGTCCCTCGGTAATGGCGAATGGGACCCAGAACTCTCCTTAGCTTCCNAAAGAGAAGCAGGAGAAAACTGGCTCTCCCTTAGCCATCCGAGT<br>GGACGTCTGTCCTCCTTCGGATGCCCAGGTCGGACCGCGGGGAGGTGGAGATGCCATGCCGACCCGAAG-AGGAAG—AAGAGGACACGGACGC<br>GAACCGTGAGTGGAACCCTCGATCCTTTATTGGGGGGTACACTCGAGGAGTGGAAGGCGGGGAGAGGGGCGCCCAGGGTCAACCTACGGAAATCC<br>CTGAGTTCCTCTGATGTCCAGGCCGTCCCCTCTTCTAGAGAAGGGAGACTCCGGAACTCCTTCCATGGTTGGGACGAAGCCGCCNCCGGGCGCTCC<br>CCTCGGAGGTCCCTCGAGGGGGTTCACATCCCCAACCCGCGGGCCGGCTACTCTTCTTTCCCTTCTCTCGTCTTCCTCGGTCAACCTCCGAAGTTCC<br>TCTTCTTCCTCCCTGCTGAGGNTCTTTCCTCCGGAGGAGAGCTGCTTTTTCTTGTTCTCCAGGGCCTTCCTTCTTCGGTGGTCCCGCCTCTCCTGTTCG<br>GTGAACCCTCCCGGGTGTTTCCTCTTCCTAGGTCCGGAGTCGACCTCCATCTGATCCGTCCTGGCTCTCTTCGCCGGGGGAGCTCCCTCCCCATCCT<br>TGTCCTTTCTTATTATTCCTCGGATGTTCCCCAGCCAGGGATTGTCCTCCTCNAGTCTCTTGAGATTCTTGTTGACCTTCCGGAGCTCCCTCTCGAGTT<br>CCTCCTTTCTTCTTCTTCCATCGATCCACTTCCCGAGTGTCTCTTCTCTCCCCCTCCGGGGTCTCCTCGNATCGGACTGGCTCATCCTCGA--<br>AGAGGGNGGGCGGTCCTGAGAACTCTTATCTTCCTGCTTAGAAGAGGAGTCTCCTGGACG-CNCCCGCCCGNTCGG-
**5**	ATGGGCCNCNGTGCCGACGAAGGNCCGGAGGAG—GAGAGGNNAGGGNNCCCGAGNGGGGATGCAAGCNAAAGAGTGGANGANTCNTCGGGGAA<br>NN-NNCTCCCAAGAAGAACCGGGAN-CNATCTCAAGAAGAGGA-G-N—TTTCCCAAACGCNGGTGGAACNCCCGGGAACAAGGAAACAGGAGAAGGA<br>ACGGTAGAAAAGAGCGA-GCCCTCCGATCCGANGGGGCCCTGGACNCCTCAGCTCTGGAGTCCTC-CGACCTGTGAGGTCGAGGAT-CCCCG-CGCA<br>GGG-AGACAGCCACCAGGAGAGAA-CAGAGAAATCACCTC-CAG-AGGACCCCTTCA-GCGAACAATGAAGCGCNCTGAGNCG-TCAGGA-TGAGNCCA<br>TAGCGATAGGAGGGGATGCTAGGAGTCGGCGGCGACCG--AAGCGAGGAGGAAAGN-AAAGAAAGCAACGGGGCTAGCGAGTGGATGTTCCGCCTC<br>CCCGNGGGTCCCGAGTGAGGCTTATCCCGGGGA-ACTCGGCCAAG-TCCCGAGATGNCTGGTT-CCACNGTCTCCACTTCC-AAAAGGAGGA-GGGGG<br>GGGACTTGGAGCANG-GGGG-CCAGTCCAGTCCGTGGGAATTACCCCGGANCTCCCGTTCTCCACACTCCTT-CCCCCCGCGGGCTCC-CCCAATAAA-<br>ANNGGAAGGACCCA-CCCACTGGGGTCCGACGTTCCNACCCTTTCTTACC-TGTGGCCGGCATGGTCCCAGCCTCCTCGCTGGCGCCGGCTGGGCAA<br>CATTCCGAGGGGACCGT-C-CCTCGGTAATGGCGAATGGGACCCAGAACTCTCTCTAGATTCCCAGAGAGAATCGAGAGAAAACTGGCTCTCCCTTAG<br>CCATCCGAGT-GGA-CGTT-NGTCCTCC-TTCGGATGCCCAGGTCGGACCGCGGGGAGGTGGAGATGCCATGCCGACCCGAAG-AGGAAAGAAGGACT<br>CGGACGCGAACCCGTGAGGTGAACCTTTN—TCCTTTATTGGGGAGTACACTCGAGGAGTGGAAGGCGGGGAGGCGGGGNTCCCGGGCTTACCCAC<br>GGGAACTGCTGGTTTCCCCTNATGTCCAGTCCCTCCCCCGTCCTGGAGAAGGGAGACTCGGGAACGCTTAGCATCTGAGGGACAAAGCCGCCCCCG<br>GGCGCTCCCCTCGGAGGTCCNCCGGGTGGGTTCACATCCCCAACCCGCGGGCCGGCTACTCTTCTTTCCCTTNTCTCGTCTTCCTCGGTCAACCTCC<br>GGAGTTCCTCTTCCTCCTCCTTGCTGAGTGNCTTTCCTCCCGCGCTCAGCTGNTTCTTCTTGTTCTCGAGGGCCTTCCTTCTTCGGTGATCCTGTCTCT<br>CCTTGTCGGTGAAGTCTCCCGCGCGNGTCCTCTTCCTAGGTCCGGAATCGATCTCCATCTGATCCGTCCGGGCCCTCTTCGNCGGGGGAGCTCCCTC<br>CCCGTCCTTCCCTTTTCTTATTATTCCGAGGATGTTCCCCAGCCAGGGATTTTCATCCTCGAGTTTCTTGATGGTTCTCCTGGTCTTCCGGAGCCTTCT<br>CTCGAGATCCTCCGCATCCTTTCTTGCTTGNACCCACTTNTCGAGGATNTCCTCCCTNCCTCCCCTCCGGGATTTCTTCTGNTCGGACTGGCTCATCTT<br>CGGN-AANGGGGGCGGCGGC-GTCTGTTCTCTANTCTTTTCT--TTNGAAAGAGGAGACAGCTNGCCGCCC-GCCCCCNGCCCCGAG
**6**	-ATGGGCCACAAGCCGACGAAGAGATCGAGG--ANTTGGGGAGNNGAGAGGAACCGAGG-GGGGTTCGAAC-TTAAGAAGAGAGGATCTTCGAGGAAA<br>TACTCCCAAGAAGNNCCCGGAGAGATCCAAGAGACGGAAGAN--TTTCCCCCA-TTGGTTGGGNANAGTCCCGGAACCAGGAAATGGG-AAAGAGACG<br>GTAG-AAAAGAGCGAGCCTCCCAGTTTTCGAGTAGTCCCGGACCTATCTAAATTGGAGTCATCCGACC-GTATGGAGAAAATTTCCGACAAGAGGTGAT<br>CCACTGAGGGTTTGCGGAGAAATCACCTCCAGAAGGACCCCTTCAGCGAACAGAAGAGACTGGAAGCCCCAGAGGAAGACCATAGCCATGGGGAGA<br>GATGCTAGGAGTGG-GGGGCGACCGGAACGAGGAGAAAAGTAAAGAAAGCAAC-GGGGCTAGCGAGTGGATGTTCCGCCTCCCCGGGGAGCCGAGT<br>GAGGCTTATCCCGGAGTG-CCGGGCA-AGTCCCCCCATG-CCCGTTCCACGGCTCCCTTCCANAAGGNCGGGGGTGGCCTGGAGCGTCGGGCCCC<br>AGCAGTCCGTGGAATTCACCCTCTTACCGCTT-CTCCACACTCCTTCCCCCCTGCGGGCTCCCCCCATAAGATGACGAGGACCCCACTCATCGGGG<br>TCCGCAGTCTCATCCTTTCTTACCTGATGGCCGGCATGGTCCCAGCCTCCTCGCTGGCGCCGGCTGGGCAACATTCCGAGGGGACCGTCCCTCGG<br>TAATGGCGAATGGGACCCAGAACTCTCTC-AGAATCTAGGGANANNTCCCAGATTCGAGAGAAAACTGGCTCTCCCTTAGCCATCCGAGTGGACG-<br>TCTGTCCTCCTACGGATGCCCAGGTCGGACCGCGGGGAGGTGGAGATGCCATGCCGACCCGAAGAGGAAAGAAGGACTCGGACGCGAACTCGTGA<br>GTGGACACCTCGTTCTTTATTGGGGAGTACACTCGAGGAGTGGAAGGCGGGGAGGGCGGGGAGGAGTGTTACCCNACGGGAACTGCTGGGTNCCC<br>CTGACGTCCAGACCNTCCCCCGTCCGGGAGAATGGAGACTCGGGAACGTGTAGCATGGTGGGGACGAAGCCCCCTCCGGGCGCTCCCCTCGGACT<br>TCCGCCGGGTGGGTTCACATCCCCAACCCGCGGGCCGGCTACTCTTCTTTTCCTTTGCTCGTCTTCCCCGGTCAGCCTCCCGAGTTCCTCTTCTTCTT<br>CCCTGCTGAGGCTCTTCCCTCCGGCGGCCAGTTGCTTCTTCTTGTTCTCGAGGGCCTTCCTTCGTCGGTGATCCCGCCTCTCCTCGTCGGTGAAACC<br>CGACTTGTGC-GGCTTTCCCCTAGGTCCGGAATCGACCTCCATTTG-ATCCGT-CCGGGGCTTCTTCGTTGGGGGTGCTCCCTCCCCGTCCTTTCCC<br>TTTCTTATGATTCCGANGATGTTTCCCAACCAGGGATTGTCNTCCTCTAGTTTCTTGAGACTCTTCTGGGTCTTCCGGAGCTTCCTTTCGAGATCNTCT<br>CGATTTTTTCTTAGTTCCACCCACTTCTCGAGGATCTCTTCTCTCCCACCTCGNTTTCTCTTCTGCTCGGCGGGGCCCATCTCGACT-GGGGGGCGGC<br>GTCCTCAGTACTCTC-TAC-TTTCTCAAGAAAAGAGGAGACTGCTGGTCGCCCG-CCCCCGNGTTCGAGC
**7**	-ATGGGCCAAG-TCCGACGAAGAGTCCGGAG-ATGGGAGA-GGAGNGGGANCC-CCGAAG-GGGGCAAAGACGAGAGG-GCGGAGACACT-CTGAGG<br>AGAGGACTCCCAAG-AGGACAAGGAGNGACTCAAGATCCGGGAACGAATCCCCAAGACGCCGGTGAAGC-TCTCGGAATGAGG-AAAANGGGCGG-<br>GATGGTAGAAAAGAGC-GAGCCTCCCGATCCGAG-GTTCCCCTGAACCTATTGGATCTGGAGACTC-CGA-CCTTAAGGGGTCA-AAGGTAGGACA--<br>GGGAGGCAATCCACCAGGAG-TTCTCGGANAACCCACCTCCAGAGGACCCCTTCAGCGAACAGAAGAGC---CTGTCCCGGTCGAATAGTCCCGTAGC<br>GATA-GGAGGA-GATGCTAGGAGTGGGGAGAGACCGGTGCGAGAGGAGCAAGACAAGAAAGCAACGGGGCTAGCGAGTGGATGTTCCGCCCCCC<br>GTTGGCTCCGAGTGAGGCTTATCCCGGGGAACTCGGC-TATCTTCCCCAACTAGCCGGNCCCGGATCCCCTTCCAAACGAACGG-GGGGACNN-CGG<br>CNGNNCGGGGGNCCCGCNGCNCCATGGTG-AACCCCCGTAGCCCCCCACTCCACACTCCTT-CCCCCCGCGGGGCCCCCCATCC-AAAGATGAAA<br>CTCCACCCTCCAGGGTTCGCTATCCTACCCTTTTCTTACCTTTGGCCGGCATGGTCCCAGCCTCCTCGCTGGCGCCGGCTGGGCAACATTCCGA-GG<br>GGACCGTCCCTCGGTAATGGCGAATGGGAC-CCAAGACCTCC-AAGATTCCCAGAGAGAATCGGGAGGTGACTGGGCATTCCCTTTGCCATCCGAG<br>TGGACGTNTGTCCTCCTTCGGATGCCCAGGTCGGACCGCGGGGAGGTGGAGATGCCATGCCGACCCGAAGAGGAAAGAAGGA-TTCGGACGCAAA<br>CCTGTGAGTGGAAG-TTNNCTCTTTATTGGGGGGTACACTCGAGGAGTGGAAGGCGGGGAGGGGGGGTCGGACTAGGTCCCYATGGAAACTGCT<br>GGTTTCCTCGGANGTCGAGTCCNTCTCCCGTTCTGGAGAAGGGGGACTCCGGGACTCCTAGCAGTTGAGGAACGAAGCCGCCCCCGGGCGCTCCCC<br>TCGGTGGTCCCTCGGGAGGGTTCACATCCCCAACTCGCGGGCCGGCTACTCTTCTTGCTCTTTGCTCGTCATCGACGGTCAACTTCCCGAGTTCCTCT

	TCTTCCTCCTTGCTGAGGGACTTTCCTCCCGCGGACAGCTGCTTCTTCTTGTTCTCGAGGGCCTTCCTTCGTCGGTGGTCCCGTCTCTCCTCGTCGGT<br>GAAGCCTCCCTTGTTACTCTTTTTCCCTGGTCCGGAGTCGACCTCCATCCGATCTGTTCGGGNTCTCTTCGCCGGGGGAGCTCCCTCCCCACTCCTGT<br>CCTTTCCAATTATTCCTTTGATGTTTCCCAGCCAGGGATTTTCGTCCTCAAGTTTCTTGATAGTCTTCNTTGCCTTCCGGAGCTTCCTCTCGAGGTCNTC<br>CGCNTCTTCTCTTGCTTTGACCCACTTCGAGAGGATCTCCTCCCTT---CCCTTCCGGGATCTCTTCGGATCGGCGAGGCTCATCTCGGC-AGAG<br>GCGGCGATCCTCAGTGCTCTTACTCTTTTCTTG-AAAGAGGAGACTGCTGGACGCCCCG-CCCGAG-TCCGAGC
**8**	--ATGGGCCAAGANCCGACGAAGAGGCCGAAGGTAGGAGAAGGAGGGG---CCCCCAAAGGGATTCGAAGCNAAAGAGTGGAGACACACGAGAAGG<br>ANGGTGTCCCAAGAGGGCGAGGGGAGNTCNNANNGNAGAGGANGNATCCCCGAGACGCT-GGAGACTCCCGGAACAAGGAAANNGGGGATNGGA-<br>AGGTAGAAAAGAGCGAGCCTCCCGATCCGAGTTTGCCCTGAACCTATCGAG-TTTGGAGTCCTCCGAGCTTAGGGTCGAATAGAGCGCAGNGGGA<br>GGAAGCCACCAGGAGCCGGAGACAAGACACCTCCAGAGGACCCCTTCAGCGAACAGAAGAGTCTATNCCCNG-ANGAGNNGANCNTAGCGATAGGA<br>GGAGATGCTAGGAGTAGGGAGAGACCGAAGCGAGGAGG-AAAGNAAAGANAGCANCGGGGCTAGCAGGAGGGTGTTCCGCCCCCCGGCGGGGCC<br>GAGTGAGGCTTATCCCGGGGAACTCGGNCNAGTCCCGGATTNCCGGCTCCAGGACTCCACTCC-AAAGGACCGAGGGTAGGACTTGGAGCACCGGG<br>GATCCCNN-GCAANTCCATGGTGGAC-TCCGTCCCCCTTCTCCACACTCCTTCCCCCC---GCGGGCCCCCCCNTAAAGNGGAGAACCCCACTCTNCAG<br>GGTCCGCGCCCTCCCCCNTTTCTTACCNTGTGGCCGGCATGGTCCCAGCCTCCTCGCTGGCGCCGGCTGGGCAACATTCCGAGGGGACCGTCCCC-<br>CGGTAATGGCAGAT-GGGACCCAGGC-TCTCCCCGGATTCCCTNNNGNGGATCGAGGGGN—ANAGCTGGCTCTCCCTTAGCCATCCGAGTGGACGT<br>CCGTCCTCCTTCGGATGCCCAGGTCGGACCGCGGGGAGGTGGAGATGCCATGCCGACCCGAAGAGGAAAGAAGGAGACCGGACGCAAACCTGTGA<br>GTGGAACTCTCTTCCTTTATTGGGGGGTACACTCGAGGAGTGGAAGGCGGGGAGGGGGGGGCGGNNNNTGTCCCCATGGAAANTGCTGGTTTCCCC<br>TGATGTCCAGTCCATCCCCGNGTCTGGTGAATGGAGACTCCGGAACACCTAGCATCCTAGGGACGAAGCCGCCCCCGGGCGCTCCCCTCGGACTTC<br>CTCCGGGAGGGTTCACACCCCCATCCTGCGGGCCGGCTACTCTTCTTTCCCTTNTCTCGTCTTCCTCGGTCAACTTCCTGAGTTCCTCTTCTTCCTCCT<br>TGCTGAGGNTCTTCCCTCCGGAGGATAGTTGCTTCTTCTTGTTCTCGAGGGCCTTCCTTCTTCGGTGATCCCTCCTCTCCTCGTCGGTGAATCCTCCCT<br>TGCGACTCTTCTTCCCCGGACCGGAGTCAACCTCCATCTGATCTGTTCGGGCTCTCTTCGCCGGGGGAGCTCCCTCCCCTTCCTTCCCCTTTCTTATT<br>ATTCCGANGATGTTCCCCAGCCAGGGATTNTCNTCCTCGAGTTTCTTGATGGTCCTCCTCGTCTTCCGGATCCTCCTCTCGAGGTCNTCCAGATCTTTC<br>CTTGCTTCGACCCATTTTGNGAGGGTCTCCTCCCGNCCTCCCCTTCGGNNTCTCTTCGNATCGGACTGGCTCATCTCGACGAGGGCGGCGATCCTCA<br>GTGCTCTTACTCTTTNCGANAGAAAGAGGAGACTGCTGGACGCCCCGCCCGGGTCCGAGC-

All HDV genome sequences reported through December 2023 were aligned to obtain consensus sequences for each HDV genotype. Each position reflects the nucleotide that was present most frequently at that specific site across all HDV genotypes. The threshold for collecting the consensus sequences was set to >50%. Abbreviations: HDV, hepatitis delta virus.

Supplementary table 5. Percentage identity values and sequence identifiers for the proposed complete genome HDV reference sequences per genotype.

Genotype	Reference Sequence	Percentage Identity
**1**	NCBI Reference Sequence: NC_001653.2	89.49
**1**	KJ744223.1	92.28
**2**	MZ671233.1	96.49
**3**	Reverse complementary sequence of KF786346.1	96.82
**4**	AB118822.1	96.07
**5**	LT604957.1	92.47
**6**	AJ584847.1	96.97
**7**	MG711711.1	95.57
**8**	AM183330.1	93.86

Percentage identity indicates the percentage of amino acids being identical at each position to the consensus sequences provided in Supplementary Table 2. Abbreviations: HDV, hepatitis delta virus.

Supplementary table 6. Proposed complete genome HDV reference sequences per genotype.

Genotype with Sequence Identifiers	Proposed Complete Genome HDV Reference Sequences
**1**<br><br>**KJ744223.1**	CATGGGCCATCTCCGAACGAAGATCCCGCGGGGGGCAGGATCAGCGCCCGAGAGGGGTAGATGGCAAAGAGCATTGGAACGTCGAAGGAACCACTC<br>CCAAGAAGGAAAAAGAGAAGACAAGAATCGGACGGATTCCCCATGACGCTGGAGACATCTTGGAAGGGGAAAGAAGGAAGGTGGAAAAGAAGGGGC<br>GGGCCTCCCGATCCGAGGGGCCCGACTACCAAGTTTGGAGAGCACTCCGGGCCCAAGGGTTGAGAGTACCCAGAGGGAGGAATCCACACGGAGAAA<br>AGCAGATAAATCACCTCCAGAGGACCCCTTCAGCGAACAAAGAGCTCTGACACGCGAGGAGTAAGAGCGTAGCGATAGGGGGAGATGCTAGGAGTTA<br>GAGGAGACCGAAGCGAGGAGGAAAGCAAAGAAAGCAACGGGGCTAGCCGGTAGGTGTTCCGCCTCCCGAGAGGGGACGAGTGAGGCTTATCCCGG<br>GGAACTCGGCGAATCGTCCCCACATAGCAGCTCCCAGGACCCCCTTCCAAAATGGTCCGAGGGGGGTGGCTTGGAACGCAGGGAACCAGTGGAGCC<br>ATGGGATGCTCATCCCGATGCTCGATTCCATTCCCTCCCCCCAAGGGTCGCCCAGGAATGGCGGGACCCCACTCAACTGGGGTCCGCGTTCCATCCT<br>TTCTTACCTGATGGCCGGCATGGTCCCAGCCTCCCCGCTGGCGCCGGCTGGGCAACATTCCGAAGGGGACCGTCCCTCGGTAATGGCGAATGGGAC<br>CCACAAATCTCTCTAGATTCCCAGAGAGAATCGAGAGAAAACTGGCTCTCCCTTAGCCATCCGAGTGGACGTTGTCCTCCTTCGGATGCCCAGGTCGG<br>ACCGCGAGGAGGTGGAGATGCCATGCCGACCCGAAGAGGAAAGAAGGACGCGAGACGCGAACCTGTGAGTGGAAACCCGCTTTATTCACTGGGGTC<br>GACAACTCTGGGGAGAAAAGGGAGGATCGGCTGGGAAGAGTAGATCCTATGGGAATCCCTGGCTTCCCCTTACGTCCAGCCCCTCCCCGGTCCTGGC<br>GAAGGGGGACTCCGGGACGCCTTGCATGCTGGGGACGAAGCCGCCCCCGGGCGCTCCCCTCGATCCACCTTCGAGGGGGTTCACACCTCCAACCG<br>GCGGGCCGGCTACTCTTCTTTCCCTTCTCTCGTCTTCCTCGGTCAACCTCTTAAGTTCCTCTTCTTCCTCCTTGCTGAGCCTCTTCCCTCCCGCGGTCA<br>GCTGCTTTCTCTTGTTCTCGAGGGCCTTCCTTCGTCGGTGATCCTGCCTCTCCCTGTCGGTGAACCCTCCCCTGAGAGGCCTCTTCCTAGGTCCGGAG<br>TCTACCTCCATCTGGTCCGTTCGGGCCCTCTTCGCCGGGGGAGCCCCCTCTCCATCCTTATCTTTCTTTCCGAGAATTCCTTTGATGTTTCCCAGCCAG<br>GGATTGCCGTCCTCAAGTTTCTTGATTTTCTTCTTTACCTTCCGGAGTTCCCTTTCGAGTTCCTCTAACTTCTTTCTTCCATTCACCCACTGCTCGAGAAC<br>CTCTTCTCTGCCATCGCGGTGTTTCTTCGATTCGGAACGGCTCATCTCGACAAGAGGCGGGGCTCCTCAGTTCTCTTACTCTTTTCTGTAAAGAGGAGA<br>CTGCTGGAGTCCCCGCCCAAGTTCGAG
**2**<br><br>**MZ671233.1**	ATGGGCCGCAACCGGGAGAAGAGGGCGAAGTGGGGAGGGGGAGATCCCGAGGAGGATCTCCAACTAAAGAGTGGAGGAAATCTCGGAAAGCTTCTC<br>CCAAGAAGAACTGGGACGCTCTCAAGAGGGAGGAGATTTCCCGAAAGCTGGAGTCTCCCGGAATCTCATTCAAGAAGAAGGTAACGGAGAAAGGAGC<br>GGGCCTCCCGATCCGAGTGGTCCGCGGCGTATCAAGTTTGGAGTCATCCGGACGAAGGGTTGAAACAACCCACAGTCGGGTGATCCACCAGGAGGG<br>CGGAGGAGGACTAACTTCCAGAGGACCCCTTCAGCGAACGGAAGAGCTTCCTCCTTCGGAGAGAAAGACCATAGCGATGGGGGGAGATGCTAGGAGT<br>GGGCGGCGACCGAAGCGAGGGGGAAAGTAAAGAAAGCAACGGGGCTAGCGAGTGGATGTTCCGCCCCCCGGCGGGGCCGAGTGAGGCTTATCCCG<br>GGGATCGGCGAATCGTCCCGGGATGGGGACTCCGAGAGCCCCTTCCGAAGTGACGGGGGGGGGTCACTAGGAACACGGGGGACCAGTGGATCCGT<br>GGGATTACCCTCTCCACCCTCCCAACACACTCCTTCCCCCCTGCGGGGCCCCCCCATAAGATGGCAGGAACCCACTCATTGGGGTCCGCTGTTCCATT<br>CTTTCTTACCTTGTGGCCGGCATGGTCCCAGCCTCCTCGCTGGCGCCGGCTGGGCAACATTCCGAGGGGACCGTCCCTCGGTAATGGCGAATGGGAC<br>CCAGAACTCTCTCTAGATTCCCAGAGAGAATCGAGAGAAAACTGGCTCTCCCTTAGCCATCCGAGTGGACGTTCGTCCTCCTTCGGATGCCCAGGTCG<br>GACCGCGGGGAGGTGGAGATGCCATGCCGACCCGAAGAGGAAAGAAGAACTCGGACGCGAACCGTGAGTGGAACCCTGTTCTTTATTGGGGGGTAC<br>ACTCGAGGAGTGGAAGGCGTTGTTGGGGGGGAGATGGGCGTACCTACGGGAATCCCTGGTTTCCCCTGATGTCCAGTCCCTCCCCCGTCCGAGCGA<br>AGGGGGACTCCGGAACACCTTCCATCTGAGGGACGAAGCCGCCACCGGGCGCTCCCCTCGGACCTCCTCCGGAGAGGTTCACATCCCCAACCCGCG<br>GGCCGGCTACTCTTCTTTTCCTTTCCTCGTCTTCAACGGTCAACCTCCCGAGTTCCTCTTCTTCCTCCCTGCTGAGGCTCTTTCCTCCCGAGGAGAGCT<br>GCTTCTTCTTGTTCTCGAGGGTCTTCCTTCTGCGGTGATCCTCCCTCTCCTTGTCGGTGAACCCGCTCTTGTGAGGCTTCTTCCCAGTCCCGGAGTCGA<br>CCTCCATCTGATCCGTCCGGGGCCTCTTCGCCGGGGGAGCCCCCTCCCCGTCCTTCCCTTTTCTGATTATTCCGAGGATGTTCCCCAGCCAGGGATTT<br>TCATCCTCGAGTCTCTTGATGGTCCTCCTGGCCTTCCGGAGGTCTTTCTCGAGCTCCTCCGCCTTTCTTCTTGTTGTGATCCATTTCTCGAGGGTATCCT<br>CCCTTGTTCCCCTTCGGTTTCTCTTCGACTCGGATTGGCTCATCCTCGGAGCGAGGCGAAGGTCCTCAGTTCTCTCTATTCTTTCCTTTTGAAAGAGGA<br>GACTGCTGGACCAAACGCCCGACCCGGG
**3**<br><br>**Reverse complementary sequence of antigenomic sequence KF786346.1**	ATGGGCCGCTTCCGGCGAGGGAGCCCGAAGTGATAGCGGGGAGAACTCCCGAGAGTTGGGAGACAGAAAGAGGGCGAATGGTCTCGGATGGATCCC<br>CCAAGCTGCCAACAGAGGAAGAGAACAAGATAGAGGAGCCCCCGTGACGCCGGCGAACCGTCGCGAACTGGGAGAGGAGTGGCGAAGATAGTAAAG<br>CGGAGACCCCGATCCGAATGCCCGTCGGCAGCAAATTCCTCTGGAGTCCTCCGGATCCAAAGGAGAAAACTACCGGCGGAGGGTGATCCACCCGGA<br>GTTGGACGGACAAGCCACATCCAGAGGACCCCTTCGACGAACAGAAGACCCTGGTACCGGGGAGGAATAGCCCATAGTACAAGGGGAGATGCTAGGA<br>GTCGGGGGAAGCCAGAACGACTGAGAAAGAAAAGAGAGCAACGGGGCTAGCTGCCGGGTGTTCCATCCATGGGATCGGTGCCGAGTGAGGCTTATC<br>CCGGGGGTGACGCCTCGGTCCTCCCTTAGCATCGGAGTCCCGGGCCCCCTCCCAAGAATTGGAACGAGGGGAGATCGACCGGGTCCCGCAGAACCC<br>GATGGAGTCCCCCTCCATCCCCTTTCGGATAAAACTGGTCCCGATAGGGGCAACCCACAATAGGATGGCAAAGGGGACTCTCGGGTCCGTCGTTCCAT<br>CCTTTCTTACCTTGTGGCCGGCATGGCCCCAGCCTCCTCGCTGGCGCCGGCTGGGCAACGATCCGAGGGAACTATTCCTCTCGAGAATCGGCAAATG<br>GGGCCCCTCGCTCGTATCTCCGAGAGGAGACGAGAAGGAGGTGGATCTCCCTTTGCCATCCGAGGGAACTACGTTCTCCTTACGGATGCCCAGGTCG<br>GACCGCGAGGAGGTGGAGATCCCATGCCGACCCGAAGAGGAAAAGGAGGACACGGACGACAACCGTGAGTTCTATTGCCCTTTATTGTTGGGTGCAC<br>CCTGGGACCCAGTAATACCCGGGGGGAGGCGGGGTAACCCATACATGGGAACTGCTGGGTTCCTCGGATGTCGATCCCCTCTCCCGTTCGGGAAAAG<br>GGGGACTCCGGAACTCCCTGCAGGCTGGGCACGAAGCCCCCACCGGGCGCTCCCCTCGGCGGGCCGTCCATTGGGTTCACACCCCCAGGCCGCGG<br>GCCGGCTGTTCTTCTTTCTCTTTCGTCGTCATCCTGGCCAGCCTCCGGAGTTCCTCTTCTTCCTCTTGGCTGAGGGCTTTCCCCCTCCGGCCAGTTGCT<br>TCTTCTTGTTCTCAAGGGCCTTCCTTCTTCTGTGGTCCCGCCTCTCCTGGTCGGTGAACCCCCTGGCCTTGGGTTTCCTCCCAGTCCGGAATCAACCTC<br>CATGATTTCCTGCCTGGGCCTCTTCGTCGGGGGCGCTCCGTCTTCGTCCTTCTTCCTTCTCAACAGTCCGACGACGTTTCCCAGCCAGGGGTTTTCATC<br>CTCAAGTTTCTTGATCTTCTTGTTGGTTCGCCGGAGATCCTTCTCGAGTATTCGCCTTTTCTTCCTTTCTTCTACCCACTGTTCGAGGATCTCCTCCCTGT<br>CCTTCGCGGTCGGCCTCGCGACGGGTTGGCTCATCCTGAGAGCGGGGAGCCTCGACGATCTCTCTAATCTCCTGAAGGAGGAAGGAGCTCTCGAGC<br>GCCCCCCCGTCTCCTCGGAT

**4**<br><br>**AB118822.1**	GATGAGCCGCAGTGCCGACGAGGAGTCCGCAGGTGGGAGGATCAGCCACCGGAGAGGGACGCGATGGCAAGAGTGGAGGAAAGTTCGGAGGGAAA<br>TCCCAAGAAGGTCACTCGAGATTCAAGAGGTGAGGAGGGATCCCCGAGACGCTGGAGGAACACCGGGAAAGAGAAAAGAAGGCAAGAGATCGGTAG<br>AAAAGAGCGAGCCTCCCGAAACGAGTTTGCCAGGACCTATCAAGTTTGGAGTCATCCGGCCCGTAGGGGAGAATAGAACACCGGGGGGTGATCCACC<br>AGGAGAAGTAGCGGAGAACCCACCTCCAGAGGACCCCTTCTGCGAACAGAAAAGCTCTTCCCCCTCGGGAGTGGTCCCGTAGCGATGGGAGGGGAT<br>GCTAGGAGTTGGGAGAAACCGAAGCGAGGAGGAAAGCAAAGAAAGCAACGGGGCTAGCGAGTGGATGTTCCTCCCCCCGAGTGGTCCCGAGTGAGG<br>CTTATCCCGGGGATCGGCCTCCGTCCCTCCATGGTGGACTCCAGGACCCCCGAAAGGAGGGGGGTGGGCCTTGGACCTCCGGGAGCCATGGGATCC<br>GTGGAGTACCCGGGCCATCCCCTTCTCCACACTCCTTCCCCCCTAACGGGGCCCCCCCATAAGATGGCGAGAAATCCACTCATGGGTCCGTCGTCCAT<br>CTCTTTCTTACCTTTTGGCCGGCATGGTCCCAGCCTCCTCGTTGGCGCCGGCTGGGCAACATTCCGAAGGGGACCGTCCCTCGGTAATGGCGAATGG<br>GACCCAGAACTCTCTTTAGCTTCCAAAAGAGAAGCAAGAGAAAACTGGCTCTCCCTTAGCCATCCGAGTGGACGTCTGTCCTCCTTCGGATGCCCAGG<br>TCGGACCGCGAGGAGGTGGAGATGCCATGCCGACCCGAAGAGGAAGAAGAGGACACGGACGCGAACCGTGAGTGGAACCCTCGATCCTTTATTGGG<br>GGGTACACTCGAGGAGTGGAAGGCGGGGAGAGGGGCGCCCAGGGTCAACCTACGGAAATCCCTGTGTTCCTCGGATGTCCAGGCCGTCCCCTCTTC<br>TAGAGAAGGGAGACTCCGGAACTCCTTCCATGATTGGGACGAAGCCGCCGCCGGGCGCTCCCCTCGGAGGTCCCTCGAGGGGGTTCACATCCCCAA<br>CCCGCGGGCCGGCTACTCTTCTTTCCCTTCTCTCGTCTTCTTCGGTTAACTTCCGAAGTTCCTCTTCCTCCTCCCTGCTGAGGGACTTTCCCCCGGAGG<br>AAAGCTGCTTTTTCTTGTTCTCCAGGGCCTTCCTTCTTCGGTGGTCCCGCCTCTCCTGTTCGGTGAACCCTCCCGGGTGTTTCCTCTTCCTAGGTCCGG<br>AGTCGACCTCCATCTGATCCGTCCTGGCTCTCTTCGCCGGGGGAGCTCCCTCCCCGTCCTTGTCCTTTCTTATTATTCCTCGGATGTTCCCCAGCCAGG<br>GATTGTCCTCCTCGAGTCTCTTGAGATTCTTGTTGACCTTCCGGAGCTCCCTCTCGAGTTCCTCCTTTCGTCTTCTTCCATCGATCCACTTCCCGAGTGT<br>CTCTTCTCTCCCCCTCCGGGTTCTCCTCGCATCGGACTGGCTCATCTTCGAAGAGGGCGGACGGTCCAGAGAACTCTTATCTTCCTGCTTAGAAGAGG<br>AGTCTCCTGGACGCTTCCGCCCACTCGG
**5**<br><br>**LT604957.1**	ATAGCCAAGTTGCCGAGGAAGAGCCGGAGGAGGAGAAGGAGGAACCCCGAGGGGGATGCAAGCTAAGAGTGGAGGATTTTTCGGAGAAGGGCCCCC<br>AAGAAGGTCCAGGAACACCTCAAGAAGAGGAGATTTCCCAAACGCCGGTGGAACCCCGGGAACAAGGAAACAGGAGAAGGATCGGTAGAAAAGAGCG<br>AGCCCTCCAATTCGATGGGGCCCTGGACCCCTCAGCTCTGGAGTCATCCGACCTGTGAGGTCGAGGATCCCCCGTGGAGGGAGACAGCCACCAGGA<br>GAGAGCAGAGAAATCACCTCCAGAGGACCCCTTCAGCGAACAATGAAGCGCTCTGAGCCGTCAGGGCAAGACCATAGCGATAGAAGGGGATGCTAGG<br>AGTCGGCGGCGACCGAAGCGAGGAGGAAAGCAAAGAAAGCAACGGGGCTAGCGAGTGGATGTTCCGCCTCCCCAGGGCCCCGAGTGAGGCTTATCC<br>CGGGGAACTCGGCCAAGTCCCGAGATAGCTACTCCCACGGTCTCCACTTCAAAAAGGGACTGGGGGGGGTCCTTGGAGCATGGAGGCCAGTCCAGT<br>CCGTGGGAACTACCCCGGATCTCCCGTTCTCCACACTCCTTCCCCCCGCGGGCTCTCCCAATAAAAAGGAAAGAACCACCCCACTGGGGTCCGACGT<br>TCCCACCCTTTCTTACCTGTGGCCGGCATGGTCCCAGCCTCCTCGCTGGCGCCGGCTGGGCAACATTCCGAGGGGACCGYCCCTCGGTAATGGCGAA<br>TGGGACCCAGAACTCTCTCTAGATTCCCAGAGAGAATCGAGAGAAAACTGGCTCTCCCTTAGCCATCCGAGTGGACGTCCGTCCTCCTTCGGATGCCC<br>AGGTCGGACCGCGGGGAGGTGGAGATGCCATGCCGACCCGAAGAGGAAAGAAGGACTCGGACGCGAACCCGTGAGGTGAAACTTTCACATTTATTG<br>GGGAGTACACTCGAGGAGTGGAAGGCGGGGAGGCGGGGATCCCGGGCTTACCCACGGGAACTGCTGGTTCCCTCTTATGTCCAGTCCCTCCCCTGT<br>CCTGGWGAAGGGAGACTCGGGGACGTTGAGCATCTGAGGGACAAAGCCGCCCCCGGGCGCTCCCCTCGGAGGTCCACTGGGYGGGTTCACATCCC<br>CAACCCGCGGGCCGGCTACTCTTCTTTCCCTTTTCTCGTCTTCCTCGGTCAACCTCCGGAGTTCCTCTTCCTCCTCCTTGCTGAGGYTCTTTCCTCCCG<br>CGCTCAGCTGCTTCTTCTTGTTCTCGAGGGCCTTCCTTCTTCGGTGATCCTGTCTCTCCTTGTCGGTGAAGTCTCCCGCACGGGTCCTCTTCCTAGGTC<br>CGGAATCAACCTCCATCTGATCCGTCCGGGCCCTCTTCGTCGGGGGAGCTCCCTCCCCGTCCTTCCCTTTTCTTATTATTCCGAGGACGTTCCCCAGC<br>CAGGGATTTTCATCCTCGAGTCTCTTGATGGTTCTCTTGGTCTTCCGGAGCTTTCTCTCGAGATCCTCCGCATCTTTTCTTGCTTGTACCCACTTTTCGA<br>GGGTCTCCTCCCGACCTCCCCTCCGGGATTTCTTCTGATCGGACTGGCTCATCTTCGGAAAAGGGGGCGGCGGCGTCCGTTCTCTATTCTTTTCTCTA<br>GAAAGAGGAGACGGGTCTCCGCCCGCCCCCAGCTCCGAG
**6**<br><br>**AJ584847.1**	AGTGGGCCACAAGCCGGCGGAGAGATCGAGGATTTGGGGAGGAGAGGGGAACCGAGGGGGGTTCGAACTCAAGAAGAGAAGATTTTCGAGGAAATA<br>CTCCCAAGAAGTTCCCGGAGGGATCCAAGAGACGGAYGACTTTCCCCYATTGGTTGGGAAAAGTCCCGGAACCAGGAAATGGGAAAGAGACGGTAGA<br>AAAGAGCGAGCCTCCCAGTTTTCGAGTAGTCCCGGACCTATCTAAATTGGAGTCATCCGACCGTATGGAGAAAATTTCCGACAAGAGGTGATCCACTGA<br>GGGTTTGCGGAAAAATCACCTCCAGAGGACCCCTTCAGCGAACGGAAGAGACTGGAAGCCCCAGAGGAAGACCATAGCCATGGGGAGAGATGCTAG<br>GAGTGGGGGGCGACCGGAACGAGGAGAAAAGTAAAGAGAGCAACGGGGCTAGCGAGTGGATGTTCCGCCTCCCCGGGGAGCCGAGTGAGGCTTAT<br>CCCGGAGTGCCGGGCAAGTCCCCCCATGCCCGTTCCACGGCTCCCTTCCAGAAGGGCGGGGGTGGCCTGGAGCGTCGGGCCCCAGCAGTCCGTGG<br>AATTCACCCTCTTACCGCTTCTCCACACTCCTTCCCCCCTGCGGGCTCCCCCCATAAGATGACGAGGACCCCACTCATCGGGGTCCGCAGTCTCATCC<br>TTTCTTACCTGATGGCCGGCATGGTCCCAGCCTCCTCGCTGGCGCCGGCTGGGCAACATTCCGAGGGGACCGTCCCTCGGYAATGGCGAATGGGACC<br>CAGAACTCTCTCAGAATCTAGGGAGATCTCCCAGATTCGAGAGAAAACTGGCTCTCCCTTAGCCATCCGAGTGGACGTCTGTCCTCCTACGGATGCCC<br>AGGTCGGACCGCGGGGAGGTGGAGATGCCATGCCGACCCGAAGAGGAAAGAAGGACTCGGACGCGAACCCGTGAGTGGAAACCTCGTTCTTTATTG<br>GGGAGTACACTCGAGGAGTGGAAGGCGGGGAGGGCGGGGAGGAGTGTTACCCYACGGGAACTGCTGAGTGCCCCTGACGTCCAGACCCTCCCCCG<br>TCCGGGAGAATGGAGATTCGGGAACGTGAAGCATGGTGGGGACGAAGCCCCCTCCGGGCGCTCCCCTCGGATCTCCGCCGGGTGGGTTCACATCCC<br>CAACCCGCGGGCCGGCTGTTCTTCTTTTCCTTTGCTCGTCTTCCCCGGTCAGCCTCCCGAGTTCCTCTTCTTCTTCCCTGCTGAGGTTCTTCCCTCCGG<br>CGGCCAGTTGCTTCTTCTTGTTCTCGAGGGCCTTCCTTCGTCGGTGATCCCGCCTCTCCTCGTCGGTGAAACCCGACTTGTGCGGCTTTCCCCTAGGT<br>CCGGAATCGACCTCCATTTGATCCGTCCGGGCCTTCTTCGTTGGGGGTGCTCCCTCCCCGTCCTTTCCCTTTCTTATGATTCCGATGATATTCCCCAAC<br>CAGGGATTGTCATCCTCGAGTTTCTTGAGGCCCTTCTGGGTCTTCCGGAGCTTCCTTTCGAGATCCTCTCGATTTTTTCTTAGTTC<br>CACCCACTTCTCGAGGATCTCTTCCCTCCCACCTCGTTTTCTCTTCTGCTCGGCGGGGCCCATCTCGACTGGGGGGCGGCGTCCTCAGTACTCTCTTA<br>CTTTCTCAAGAAAAGAGGAGACTGCTGGTCGCCCGCCCCCGTGTTCGAG

**7**<br>**MG711711.1**<br>Sequence in standardized order	ATGGGCCAAGTCCGACGAAGAGTCCGGAGATGGGAGTGGAGTGAAAACCCCGAGGAGGCAAAGTCGAGAGAGCGGAGACACTCTGAGGAGAAGACC<br>CCCAAGAAGACAAGAAGTGACTCAAGATCCGGGAACGGTTCCCCAAAACGCCGGTGAAGCTCTCGGAATGAGGAAAATGGGCGGAATGGTAGAGAAG<br>AGCGAGCCTCCCGATCCGAGGATTCCCTGAACCTATTGGATCTGGAGACTCCGACCCTAAGGGGTCAAAGGAAGGGCAGGGAGGCAATCCACCAGGA<br>GTTCTCGGAAAACCACCTCCAGAGGACCCCTTCAGCGAACAGAAGAGCCTATCCCGGTCGAACAGTCCCGTAGCGATAGGGGGAGATGCTAGGAGTA<br>GGGAGAGACCGGTGCGAGAGGAGCAAGACAAGAAAGCAACGGGGCTAGCGAGTGGATGTTCCGCCCCCCGTTGGCTYCGAGTGAGGCTTATCCCGG<br>GGAACTCGGCTATCTTCCCCAACTAGCCGGCCCCGGATCCCCTTCCAAACGATCGGAGGGGACCTCGGCAGCGCGGGACCCGCAGCTCCATGGTGA<br>ACCCCCGCAGCCCCCCMCTCCACACTCCTTCCCCCCGCGGGGCCCCCCGTCCAAAGATGGAACTCCACCCTCCAGGGTTCGCTATCCTACCCTTTTC<br>TTACCTTTGGCCGGCATGGTCCCAGCCTCCTCGCTGGCGCCGGCTGGGCAACATTCCGAGGGGACCGTCCCTCGGTAATGGCGAATGGGACCCAAG<br>ACCTCCATGATTCCCAAAGAGAATCGGGAGGTGACTGGGCATTCCCTTTGCCATCCGAGTGGACGTCTGTCCTCCTTCGGATGCCCAGGTCGGACCGC<br>GGGGAGGTGGAGATGCCATGCCGACCCGAAGAGGAAAGAAGGATTCGGACGCAAACCTGTGAGTGGAAGTTCACTCTTTATTGGGGGGTACACTCGA<br>GGAGTGGAAGGCGGGGAGGGGGGGTCGGACTAGGTCCCYATGGAAACTGCTGGTTTCCACGGATGTCGAGTCCATCTCCCGATCTGGAGAAGGGGG<br>ACTCCGGGACTCCTAGCAGTTGAGGAACGAAGCCGCCCCCGGGCGCTCCCCTCGGTGGTCCCTCGGGAGGGTTCACATCCCCAACTCGCGGGCCGG<br>CTACTCTTCTTGCTCTTCGCTCGTCATCGACGGTCAACTTCCTAAGTTCCTCTTCTTCCTCCTTGCTGAGGGACTTTCCTCCTGCGGATAGCTGCTTCTT<br>CTTGTTCTCGAGGGCCTTCCTTCGTCGGTGGTCCCGTCTCTCCGCGTCGGTGAAGCCTCCCTTGCTACTCTTTTTCCCTGGTCCGGAGTCGACCTCCA<br>TCCGATCTGTTCGGGATCTCTTCGCCGGGGGAGCTCCCTCCCCATTCTTGTCCTTTCCAATTATTCCTTTGATGTTTCCCAGCCAGGGATTCTCGTCCTC<br>TAGTTTCTTGATAGTCTTCTTTGTCTTCCGGAGCTTCCTCTCGAGGTCTTCCGCTTCATCTCTTGCTTTGACCCACTTCGAGAGGATTTCCTCCCTTCCCT<br>TCCGGGATCTCTTCGCATCGGCGTGGCTCATCTCGGCAGAGGCGGCGATCCTCAGTACTCTTACTCTTTTCTTGAAAGAGGAGACTGCTGGACGCCCC<br>GCCCGAGTCCGAGC
**8**<br>**AM183330.1**	ATGGGCCACGAACCGACGAAGAGTCCGAAGGTAGGAGAAGGAGGGGCCCCCAAAGGGATTCGAAGCTAAAGAGTGGAGACACACGAGAAGGGAGGT<br>GTCCCAAGAGGGCGAGGGGAGGTCTCAAGGAGAGGAGGGATCCCCGAGACGCTGGAGACTCCCGGAACAAGGAAAGTGGGGATAGGAAGGTAGAA<br>AAGAGCGAGCCTCCCGATCCGGGATTGCCCTGAACCTATCGAGTTTGGAGTCCTCCGAGCWYAGGGTTGAATGGAGCGCAGGAGAGGGAAGCCACC<br>AGGAGCCGGAGACAAGACACCTCCAGAGGACCCCTTCAGCGAACAGAAGAGTCTATCCCCAGATGAGAGGACCGTAGCCGAGGGGGAGATGCTAGG<br>AGTAGGGAGAGACCGAAGCGAGGAGGAAAGYAAAGAAAGCAACGGGGCTAGCAGGAGGGTGTTCCGCCCCCCGGCGGGGCCGAGTGAGGCTTATC<br>CCGGGGAACTCGGACCAGTCCCGGACTGCCGGCTCCAGGAATCCGTTCCAAAGGACCGAGGGTAGGACTTGGAGCACCGGGGATTCCARGCAAACC<br>CATGGTGGACTCCGTCCCCCTTCTCCACACTCCTTCCCCCCGCGGGCCCCCCATAAAATGGAGAACCCCACTCTACAGGGTCCGCGCCCTCCCCCT<br>TTCTTACCTGTGGCCGGCATGGTCCCAGCCTCCTCGCTGGCGCCGGCTGGGCAACATTCCGAGGGGACCGTCCCCTCGGTAATGGCAGATGGGACC<br>CGAGCTTCCCCCGGATCCTCTCTTGGGGATCGAGGGGGAAAAAGCCGGCACTCCCTTAGCCATCCGAGTGGACGTCCGTCCTCCTTCGGATGCCCAG<br>GTCGGACCACGGGGAGGTGGAGATGCCATGCCGACCCGAAGAGGAAAGAAGGAGACCGGACGCAAACCTGTGAGTGGAACTCTCTTCTTTTATTGGG<br>GGGTACACTCGAGGAGTGGAAGGCGGGGAGGGGGGGGCGGACTCTGTCCCTATGGAAATTGCTGGTTTCCCCTGACGTCCAGTCCATCCCCGTGTC<br>TGGAGAACGGAGACTCCGGAACACCTAGCATCCTAGGGACAAATCCGCCCCCGGGCGCTCCCCTCGGACTTCCTCCGGGAGGGTTCACACCCCCATC<br>CTGCGGGCCGGCTACTCTTCTTTCCCTTCTCTCGTCTTCCTCGGTCAACTTCCTGAGTTCCTCTTCTTCCTCCTTGCTGAGGCTCTTCCCTCCGGAGGA<br>TAGTTGCTTCTTCTTGTTCTCGAGGGCCTTCCTTCTTCGGTGATCCCTCCTCTCCTCGTCGGTGAATCCCCCCCTGCGACTCTTCTTCCCCGGACCGGA<br>GTCAACCTCCATCTGATCTGTTCGGGCTCTCTTCGACGGGGGAGCTCCCTCCCCTTCCTTCCCCTTTCTTATTATTCCGAGGATGTTCCCCAGCCAGGG<br>ATTGTCCTCCTCGAGTTTCTTGATGGTCCTCCTCGTCTTCCGGATCTTCCTCTCGAGGTCCTCCAGATCTTTCCTTGCTTCGACCCATTTTGTGAGGGTC<br>TCCTCCCGTCCTCCCCTTCGGTTTCTCTTCGAATCGGACTGGCTCATCTCGGCGAGGGCGGCGATCCTCAGTGCTCTTACTCTTCACGATAGAAAGAG<br>GAGACTGCTGGATGCCCCGCCCGGGTCCGAGC

Abbreviations: HDV, hepatitis delta virus.

Supplementary table 7. Consensus sequences of L-HDAg protein per HDV genotype.

Genotype	Consensus sequences of L-HDAg
**1**<br>*W* - stop codon site replaced by W	-MSRSESK-KNRGGREEVLEQWVNGRKKLEELERDLRKVKKKIKKLEDDNPWLGNIKGILGKKDKDG<br>EGAPPAK-RARTDQ---MEVDSGPRKR---------PLRGGFTDKER-----QDHRRRKALE-----NKKKQLSA<br>GGKNLSKEEEEELKRLTEEDERRERRVAGPPVGGVNPLEGGSRGAPGGGFVPSMQGVPESPFTRG<br>EGLDVRGNRGFP*W*DILFPSDPPFSPQSCRPQ
**2**	MSQSESRRSRRGGREEILEKWITTRRKAEELEKDLRKARKTIKRLEDENPWLGNILGIIRKGKDGEGA<br>PPAKRARTDQMEVDSGTGKRPHKSGFTDKEREDHRRRKALENKKKQLSSGGKSLSREEEEELGRLT<br>VEDEERKRRVAGPRVGDVNPPGGGPRGAPGGGFVPQMEGVPESPFTRTGEGLDIRGNQGFPWV<br>NPSPPQQRLPLLECTPQ
**3**<br>*X* - S/N<br>*W* - stop codon site replaced by W	MSQTVAKLSSKDREEILEQWVEERKKRRILEKDLRRTNKKIKKLEDENPWLGNIVGLLR-RKKDEDGG<br>PPAKRPRQETMEVDSGPGRKPKAR-GF--T-DKERRDH-RRRKALENKKKQLAGGGKHLSQEEEEEL<br>RRLAKDDDERERRVAGPRPGGVNPMDGPPRGAPGGGFVP*X*LQGVPESPFSRTGEGIDIRGTQQFP<br>WYGFTPPPPGYY*W*VPGCTQQ
**4**	MSQPDSRRPRRGREEQLGKWIDARRRKEELERDLRKVNKTIKRLEEDNPWLGNIRGIIRKDKDGEGA<br>PPAKRARTDQMEVDSGPRKRKHPGGFTEQERRDHRRRKALENKKKQLSSGGKNLSREEEEELRR<br>LTEEDERRE RRVAGPRVGDVNPLDGGPRGAPGGGFVPSMHDVPESPFTRRGDGLDVRGVQEFP<br>WVSPQPPPPRLPLLECTPQ
**5**	MSQSDQKKSRRGGREEILEKWVQARKDAEDLERRLRKTKRTIKKLEDENPWLGNILGIIRKGKDGEG<br>APPAKRARTDQMEIDSGPRKRTRAGDFTDKERQDHRRRKALENKKKQLSAGGKSLSKEEEEELRRL<br>TEEDERRERRVAGPRVGDVNPPEGPPRGAPGGGFVPQMLGVPESPFSRTGEGLDIRGNQQFPWVS<br>PGSPPPRLPLLECTPQ
**6**	-MGPAEQKRKRGGREEILEKWVELRKNREDLERKLRKTQKSLKKLEDDNPWLGNILGIIRKGKDGEG<br>APPTKKPRTDQMEV-DSGPRGKPHKSGFTDEERRDHRRRKALENKKKQLAAGGKSLSREEEEELGR<br>LTGEDEQ RKRRVAGPRVGDVNPPGGSPRGAPGGGFVPTMLLVPESPFSRTGDGLDVRGTQQFP<br>WGNTPPRPPRLPLLECTPQ
**7**<br>*X* - S/N<br>*W* - stop codon site replaced by W	MSLADPKRSRKG-REEILSKWVKAREEADDLERRLRKARKTIKKLEDENPWLGNIKGIIGKDGSGEGA<br>PPAKRPRTDRMEVDSGPGKKSNKGGFTDEERRDHRRRKALENKKKQLSAGGKSLSKEEEEELRKLT<br>VDDE*X*RARRVAGPRVGDVNPPEGPPRGAPGGGFVPQLLGVPESPFSRTGDGLDVRGNQQFP*W*GP<br>SPTPPPRLPLLECTPQ
**8**<br>X^{1} - P/A<br>X^{2} - E/S	MSQSDX^{1}RX^{2}RGGREETLTKWVEARKDLEDLEKRIRKTRRTIKKLEDDNPWLGNILGIIRKGKEGEGAP<br>PAKRARTDQMEVDSGPGKKSRKGGFTDEERRDHRRRKALENKKKQLSSGGKSLSKEEEEELRRLT<br>EEDERRERRAAGPQDGGVNPPGGSPRGAPGGGFVPRMLGVPESPFTRTGDGLDIRGDQQFPWG<br>QQPPPPRLPLLECTPQ

All L-HDAg sequences published until December 2023 were aligned to identify consensus sequences for each HDV genotype. Each position reflects the amino acid present at the highest

frequency at that specific site across all HDV genotypes. The threshold for collecting the consensus sequences was set to >50%. Abbreviations: L-HDAg, large hepatitis delta antigen; HDV, hepatitis delta virus

Supplementary table 8. Variations in amino acid positions of the L-HDAg within conserved $CD8^+$ T-cell epitopes.

$CD8^+$ T-Cell Epitope Position	Genotype	Sequence Type	Sequences	IC_{50} (nM) and Percentile Rank with MHC-I Allele	
				IC_{50}	Rank
				**HLA-B*18:01**	
**L-HDAg$_{46-54}$**		**Published**	DENPWLGNI	767.92	0.33
		**Reference**	··H······	724.36	0.31
	1	**Proposed**	·G·······	31936.82	30
	2		·········	767.92	0.33
	3		········V	752.66	0.32
	4		ED·······	25971.33	17
	5		········V	752.66	0.32
	6		·D·······	18466.23	7.8
	7		·········	767.92	0.33
	8		ED·······	25971.33	17
				**HLA-B*27:05**	
**L-HDAg$_{99-108}$**		**Published**	RRDHRRRKAL	219.43	0.52
		**Reference**	·Q········	2451.4	2.9
	1	**Proposed**	·Q········	2451.4	2.9
	2		·E······T·	12757.56	12
	3		··········	219.43	0.52
	4		··········	219.43	0.52
	5		·Q········	2451.4	2.9
	6		··········	219.43	0.52
	*7		··········	219.43	0.52
	8		··········	219.43	0.52

				**HLA-B*37:01**	
**L-HDAg$_{100-108}$**		**Published**	QDHRRRKAL	6725.35	2.4
		**Reference**	·········	6725.35	2.4
	1	**Proposed**	·········	6725.35	2.5
	2		E······T·	12711.94	5.4
	3		R········	2125.65	0.64
	4		R········	2125.65	0.64
	5		·········	6725.35	2.4
	6		R········	2125.65	0.64
	7		R········	2125.65	0.64
	8		R········	2125.65	0.64
				**HLA-B*27:05**	
**L-HDAg$_{104-112}$**		**Published**	RRKALENK/R	143.15	0.33
		**Reference**	········R	233.2	0.54
	1	**Proposed**	········R	233.2	0.54
	2		···T····K	188.03	0.43
	3		········K	143.15	0.33
	4		········K	143.15	0.33
	5		········K	143.15	0.33
	6		········K	143.15	0.33
	7		········K	143.15	0.33
	8		········K	143.15	0.33

L-HDAg protein sequences of the proposed genomic reference sequences were compared with the published epitopes and the original NP_597693.2 reference protein. Binding affinities of epitope variants with the published MHC-I alleles were predicted *in silico* through iedb.org using the NetMHCpan BA algorithm. Binding affinity with IC_{50} values <50 nM are considered high affinity, <500 nM intermediate affinity and <5000 nM low affinity.

*For the proposed reference sequence of epitope L-HDAg$_{99-108}$ of genotype 7, the X or unknown amino acid in the first position has been replaced with the most common amino acid in that position [arginine (R)].

Abbreviations: L-HDAg, large hepatitis delta antigen; HDV, hepatitis delta virus; MHC-I, major histocompatibility complex class I; IC_{50}, half-maximal inhibitory concentration; HLA - Human Leukocyte Antigen.

Supplementary table 9. Variations in amino acid positions of the L-HDAg within non-conserved CD8⁺ T-cell epitopes.

CD8⁺ T-Cell Epitope Position	Genotype	Sequence Type	Sequences	IC50 (nM) and Percentile Rank with MHC-I Allele			
				IC50	Rank	IC50	Rank
				HLA-B*37:01			
L-HDAg$_{81-90}$		Published	VDSGPRKRPL	11617.85	4.8		
		Reference	·········S	35591.96	44		
	1	Proposed	··········	11617.85	4.8		
	2		····TG·K·H	41839.59	74		
	*3		·····GRK·K	37886.42	53		
	4		········KH	43150.34	82		
	5		········TR	40422.75	66		
	6		······GK·H	38109.66	54		
	7		·····G·KSS	39295.07	60		
	8		·····G·KSR	42199.21	76		
				HLA-B*41:01			
L-HDAg$_{140-149}$		Published	RERRVAGPPV	187.03	0.28		
		Reference	····I···Q·	478.23	0.58		
	1	Proposed	··········	187.03	0.28		
	2		·K······R·	20881.12	17		
	3		····T···RP	1107.19	1.3		
	4		········R·	732.05	0.84		
	5		········R·	732.05	0.84		
	6		·K··T···R·	21509.67	18		
	7		·A······R·	19892.23	16		
	8		········QD	11302.18	7.1		
				HLA-B*15:01			
L-HDAg$_{170-179}$		Published	SMQGVPESPF	21.93	0.07		
		Reference	··········	21.93	0.07		
	1	Proposed	··········	21.93	0.07		
	2		Q·E·······	239.44	0.69		

	3		·L········	62.0	0.23		
	4		I·E·······	123.63	0.43		
	5		Q·LN······	28.42	0.09		
	6		T·LH······	32.23	0.11		
	7		QLL·······	119.89	0.42		
	8		R·L·······	12.59	0.03		
				**HLA-B*58:01**			
**L-HDAg$_{189-196}$**		**Published**	RGSQGFPW	390.06	0.75		
		**Reference**	········	390.06	0.75		
	1	**Proposed**	········	390.06	0.75		
	2		··N·····	762.67	1.3		
	3		··T·Q···	582.99	0.96		
	4		··T·····	628.79	1.1		
	5		··N·Q···	696.62	1.2		
	6		··T·Q···	582.99	0.96		
	7		··N·Q···	696.62	1.2		
	8		··N·Q···	696.62	1.2		
				**HLA-B*35:01**			
**L-HDAg$_{192-200}$**		**Published**	QGFPWDILF	1008.23	0.76		
		**Reference**	·········	1008.23	0.76		
	1	**Proposed**	·········	1008.23	0.76		
	2		·····VRPS	22752.58	13		
	3		·Q···YG·T	34372.56	31		
	4		·····VDPG	21686.09	12		
	5		·Q···VSPG	15310.26	6.7		
	6		·Q···GNTP	25412.34	16		
	7		·Q···GPSP	24317.55	15		
	8		·Q···GQSP	24359.95	15		
				**HLA-B*35:01**		**HLA-B*07:02**	
**L-HDAg$_{194-202}$**		**Published**	FPWDILFPA	11.6	0.03	702.78	0.79
		**Reference**	·········	11.6	0.03	702.78	0.79

	1	**Proposed**	····L····	10.7	0.03	617.32	0.71
	2		···VRPS·P	412.04	0.42	482.33	0.6
	3		···YG·T·P	100.41	0.16	917.95	0.9
	4		···VDPGRP	4192.73	2.1	14589.93	7.9
	5		···VSPGSP	1009.93	0.76	2674.95	2.0
	6		···GNTP·R	301.42	0.34	3722.88	2.4
	7		···GPSPTP	394.56	0.41	2753.27	2.0
	8		···GQSP·P	127.96	0.19	1813.37	1.6

L-HDAg protein sequences of the proposed reference genomic sequences were used for comparison with the published epitopes and the original reference protein NP_597693.2. Binding affinities of epitope variants with the published MHC-I alleles were predicted in silico through iedb.org using the NetMHCpan BA algorithm. Binding affinity with IC_{50} values <50 nM are considered high affinity, <500 nM intermediate affinity and <5000 nM low affinity.

*For the proposed reference sequence of epitope L-HDAg$_{81-90}$ of genotype 3, the X or unknown amino acid in the fifth position has been replaced with the most common amino acid in that position [proline (P)].

Abbreviations: L-HDAg, large hepatitis delta antigen; HDV, hepatitis delta virus; MHC-I, major histocompatibility complex class I; IC_{50}, half-maximal inhibitory concentration; HLA - Human Leukocyte Antigen.
